# Plant hydraulic traits influencing crop production in water-limited environments

**DOI:** 10.1093/plphys/kiaf521

**Published:** 2025-10-14

**Authors:** Amanda A Cardoso, Moab T Andrade, Erika R Bucior, Samuel C V Martins

**Affiliations:** Department of Crop and Soil Sciences, North Carolina State University, Raleigh, NC 27695, USA; Department of Crop and Soil Sciences, North Carolina State University, Raleigh, NC 27695, USA; Department of Crop and Soil Sciences, North Carolina State University, Raleigh, NC 27695, USA; Departamento de Biologia Vegetal, Universidade Federal de Viçosa, Viçosa 36570-900, Brazil

## Abstract

Annual crops commonly experience production losses due to soil water limitation and increased vapor pressure deficit (VPD). Crop species and genotypes vary in their ability to sustain production during drought, which reflects variations in drought resistance mechanisms. In this review, we discuss the critical influence of water transport (hydraulic conductances and stomatal traits) on the ability of crops to avoid and tolerate drought, thus sustaining yield. We also summarize scientific gaps to be addressed in the future. Limited transpiration traits, including reduced stomatal density/conductance and increased stomatal sensitivity to soil drought and high VPD, constitute important drought avoidance mechanisms. Drought avoidance is suggested to result in soil water conservation for the critical reproductive stage and yield stability under moderate and terminal droughts. As crop fields experience increasingly drier soils and greater VPD, tolerance mechanisms might become critical to crop production. Osmotic adjustment stands as an important tolerance mechanism that improves crop production during severe droughts. Preventing xylem embolism and/or refilling embolized xylem upon rehydration represent drought tolerance mechanisms critical for plant survival during drought, but their contribution to crop production during droughts is unknown. Time for hydraulic failure combines drought avoidance and tolerance, and its importance for crop production during moderate and severe droughts should be assessed.

## Introduction

Crop production is highly dependent on soil water, which is maintained through rainfall events and irrigation. Over three-quarters of the global agricultural land remains rainfed (Rosa et al. 2020), and soil water often becomes a constraint to crop growth and yield (Mueller et al. 2012; Leng and Hall 2019). Ongoing increases in air temperature drive increases in vapor pressure deficit (VPD) and in plant water use (Grossiord et al. 2020), accelerating soil water depletion (Scheff and Frierson 2014) and exerting further challenges to crop production (Lobell et al. 2014; Kimm et al. 2020). Although global precipitation is expected to slightly increase with climate change, land aridity is projected to increase because of rises in air temperature and VPD, translating into more frequent and severe droughts (Scheff and Frierson 2014; Berg et al. 2017). Therefore, identifying traits associated with greater crop yield under drought is essential to future agriculture. Here, we describe the influence of plant hydraulic and stomatal traits on crop production in water-limited environments via drought avoidance and tolerance. We also identified scientific gaps to be addressed in the future to better understand how crop hydraulics can be manipulated to ensure crop production during drought. This review focuses on annual crops, which are largely responsible for global food production (Pimentel et al. 2012) and are often more susceptible to drought than perennials (Vico and Brunsell 2018).

## Drought resistance mechanisms and crop production during drought

**Figure 1.**
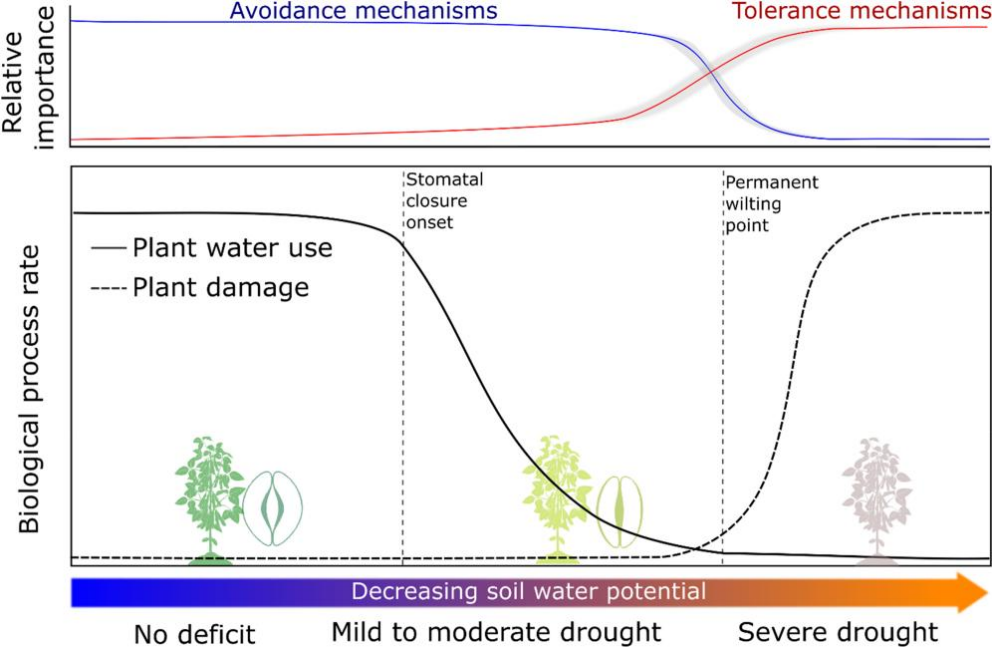
Crop physiology during drought. As soil water potentials decline, crops limit water use via stomatal regulation. After the turgor loss point, plant water use is limited to leaf residual transpiration. Turgor loss point is also known as PWP and averages −1.5 mPa for herbaceous crops excluding turfgrasses according to the dataset of Bartlett et al. (2014). Limitation to plant water use with soil drying constitutes an important mechanism of drought avoidance, effectively allowing crops to minimize tissue dehydration, conserve soil water for critical reproductive stages, and sustain production during moderate droughts (Sinclair and Muchow 2001; Sinclair et al. 2010; Zaman-Allah et al. 2011; Vadez et al. 2013; Carminati et al. 2020). Reduced residual leaf transpiration also constitutes a drought avoidance trait that minimizes plant dehydration to severe levels, thereby delaying damage to plant tissues and cells (Sinclair 2000; Duursma et al. 2019; Haverroth et al. 2023; Pereira et al. 2024). During severe droughts (post-PWP), tolerance against dehydration-induced damage (drought tolerance) becomes increasingly important. The contribution of drought tolerance traits to yield stability is uncertain, with several authors considering tolerance traits important for crop survival but not for production (Sinclair 2011; Berger et al. 2016; Vadez et al. 2024).

For crops, drought resistance means sustaining yield during drought (Vadez et al. 2024). Crop production during drought is a function of water used for transpiration, water use efficiency (WUE), and harvest index (Passioura 1977). Crops must balance water loss (via transpiration) and soil water capture to ensure high yields at the end of the season (Vadez et al. 2024). Crops can display drought resistance through 3 distinct, yet complementary, strategies: escape, avoidance, and tolerance (Box 1). Drought escape is largely influenced by phenology (Berger et al. 2016; Shavrukov et al. 2017; Sánchez-Gómez et al. 2019), while avoidance and tolerance are closely linked to the water movement throughout the soil-plant-atmosphere continuum, being influenced by plant hydraulics (Fig. 1).

Box 1. Drought resistance strategies Drought resistance can be separated into 3 strategies: drought escape, avoidance, and tolerance (Levitt 1980; Delzon 2015). The terms dehydration escape, avoidance, and tolerance are also used (Volaire 2018). While this separation is broadly accepted, some authors replace “resistance” with “tolerance” (and vice-versa), while maintaining the core separation idea (Vadez et al. 2024). In this case, they consider that drought tolerance is separated into escape, avoidance, and resistance. This last terminology is far less common.

**Drought/dehydration escape**

**Description**

Plants complete their life cycle before the onset of soil droughts or before soil droughts become severe late in the growing season.

**Associated traits and mechanisms**

Rapid plant development, early flowering, and early maturing (Shavrukov et al. 2017).

**Drought/dehydration avoidance**

**Description**

Plants minimize tissue dehydration and loss in cell turgor in drying soils.

**Associated traits and mechanisms**

1. Reductions in plant water loss and increases in intrinsic WUE (WUE_INT_) [e.g. stomatal closure with soil dehydration (Sinclair et al. 2018), lower maximum stomatal conductance (Haverroth et al. 2023), stomatal closure with increases in VPD (Sinclair et al. 2017), reductions in nighttime transpiration, reduced leaf residual transpiration (Pereira et al. 2024), reductions in total leaf area (Li et al. 2019), and leaf rolling in monocots (Wang et al. 2023)].
2. Optimized soil water capture [e.g. deeper and denser rooting systems, low root angle, long lateral roots, increases in root hair, enhanced root hydraulic conductance, maintenance of the root-to-soil contact in dry soils, and rhizosheath formation (reviewed by Vadez et al. 2024)].
3. Increased hydraulic capacitance [i.e. which minimizes declines in plant water potential for a given amount of water loss (Pereira et al. 2024)].

**Drought/dehydration tolerance**

**Description**

Plants sustain plant function at higher levels of dehydration, i.e. plants can experience lower water potentials without experiencing major damage to tissues and cells.

**Associated traits and mechanisms**

Greater xylem resistance to embolism (Cardoso et al. 2018, 2020a; Andrade et al. 2024), greater rehydration capacity (Liu et al. 2024), lower water potential associated with leaf turgor loss (intrinsic or via osmotic adjustment*) (Blum 2017; Cardoso et al. 2018), greater stability of cell membranes (Oliveira et al. 2022) and photosynthetic apparatus (Cardoso et al. 2018), and plant survival at lower water potentials (Pereira et al. 2024; Simpson et al. 2024). *Some authors consider osmotic adjustment to be a drought avoidance trait (Blum 2011; Vadez et al. 2024), while others consider it to be a drought tolerance trait (Delzon 2015; Berger et al. 2016).

Several studies demonstrate that annual crops can achieve drought resistance through avoidance—either by optimizing water capture or reducing water loss via transpiration (Manschadi et al. 2006; Blum 2011; Vadez 2014; Sinclair et al. 2017; Gleason et al. 2022; Vadez et al. 2024). While optimizing water capture through roots constitutes an important aspect of drought avoidance (Vadez 2014; Ahmed et al. 2018; Maurel and Nacry 2020; Cai et al. 2022; Li et al. 2022; Gleason et al. 2025), we focus on the “limited transpiration” aspect of avoidance in this review. Reductions in transpiration and conservation of soil water have been suggested to result not only in crop survival but most importantly in sustained yields in environments exposed to terminal droughts (i.e. when drought occurs during grain filling) (Sinclair and Muchow 2001; Sinclair et al. 2005, 2010; Zaman-Allah et al. 2011; Vadez et al. 2013; Messina et al. 2015; Collins et al. 2021). These studies argue that greater production in water-limited environments is achieved via increases in soil water availability during grain filling brought about by limited transpiration early during the season (Vadez et al. 2013; Bhatnagar-Mathur et al. 2014; Vadez et al. 2014). While this strategy has been largely linked to improved yields under terminal droughts, some studies show that it can also result in better yields under intermittent droughts (Ratnakumar et al. 2009 ; Bhatnagar-Mathur et al. 2014). It is important to note that limited transpiration often translates into lower carbon assimilation and accumulation, which can, in turn, lower the investment in deeper and denser roots that are important to avoid drought (Gleason et al. 2025).

The contribution of drought tolerance to yield stability remains uncertain. Tolerance mechanisms mitigate cellular damage under severe dehydration occurring beyond the turgor loss point (Ψ_TLP_), also known as permanent wilting point (PWP). Beyond the PWP, carbon assimilation is negligible, and growth and yield can be severely impacted if plants experience this level of drought for an extended period. Therefore, several authors consider tolerance traits important for crop survival but not production (Sinclair 2011; Berger et al. 2016; Vadez et al. 2024). However, as we improve our understanding of crop production during drought, it becomes clear that drought resistance mechanisms are crop and context specific (Tardieu et al. 2018; Gleason et al. 2022, 2025; Vadez et al. 2024). While drought avoidance is important to sustain production under moderate and terminal droughts (Vadez et al. 2024), an association between avoidance and tolerance might be critical to ensure production during more severe droughts. As climate change results in increasingly severe droughts associated with high VPD, future agriculture might eventually rely on both avoidance and tolerance acting in concert.

## Hydraulic traits contributing to drought avoidance and greater WUE

**Figure 2.**
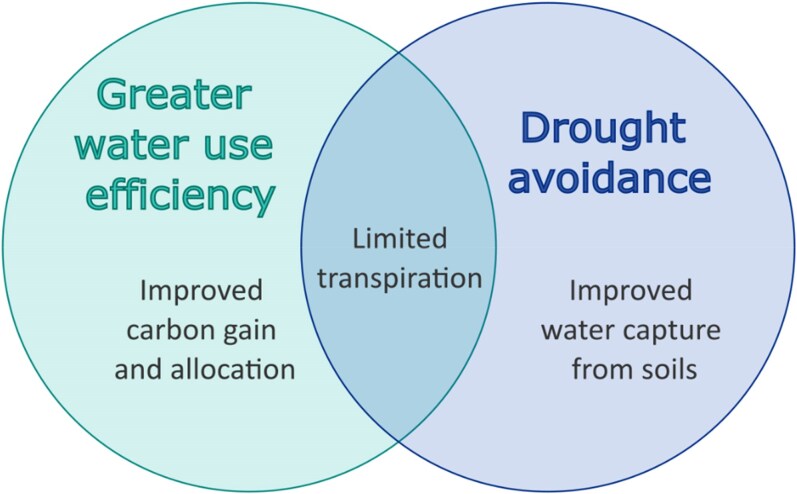
Greater WUE at the leaf and plant levels can be achieved by either increasing carbon gain and allocation, reducing transpiration, or both. Drought avoidance results from improved water capture and/or reductions in transpiration (Box 1). Traits contributing to limited transpiration can be achieved via changes in stomatal structure and regulation. They contribute to both greater WUE and drought avoidance, ultimately conserving soil water and ensuring crop yield stability in water-limited regions (Sinclair et al. 2005, 2010; Messina et al. 2015; Collins et al. 2021, but see Gleason et al. 2025).

Stomatal regulation of plant transpiration is at the intersection of drought avoidance and greater WUE (Fig. 2). Traits contributing to greater drought avoidance and WUE include high stomatal sensitivity to soil dehydration and rising VPD, as well as reduced daytime, nighttime, and residual (post-stomatal closure) transpiration. It is important to distinguish *adaptive* drought avoidance from plant responses that simply reflect soil conditions. For instance, soil type and rooting volume can shift the timing of stomatal closure independent of genotype (as happens in sandy versus loamy soils) (Koehler et al. 2022). Therefore, plant responses must be interpreted relative to the soil hydraulic context rather than as sole evidence of superior drought avoidance.

### Increased stomatal sensitivity to soil dehydration

Crops gradually reduce stomatal conductance (*g*_s_) and transpiration with declines in soil moisture, minimizing plant and soil dehydration. Besides resulting in drought avoidance, stomatal closure to soil dehydration also increases WUE. Different authors use different methods to characterize stomatal sensitivity to soil dehydration. Declines in transpiration are plotted against soil water potential (Ψ_soil_) (Théroux-Rancourt et al. 2014; Koehler et al. 2022), soil water content, or the fraction of transpirable soil water (Ray and Sinclair 1997; Sinclair et al. 1998). Other studies plot *g*_s_ against leaf water potential (Ψ_leaf_) (Skelton et al. 2017; Corso et al. 2020), given that Ψ_leaf_ declines with soil dehydration. Finally, leaf Ψ_TLP_ is also used as a proxy for the Ψ_leaf_ at complete stomatal closure (Haverroth et al. 2023; Pereira et al. 2024). Irrespective of the method, different studies demonstrate a wide variation in stomatal sensitivity to soil dehydration across crop species and genotypes (Koehler et al. 2023).

**Figure 3.**
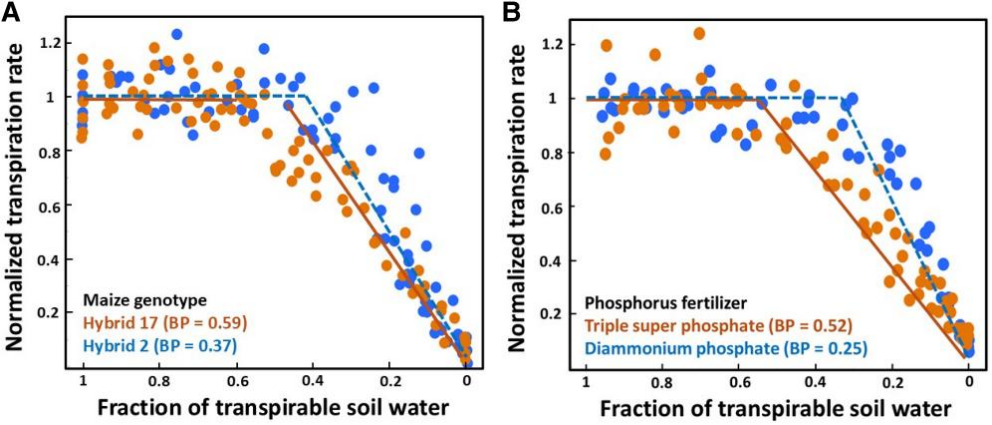
Contrasting stomatal sensitivity to soil dehydration demonstrated by declines in normalized transpiration rate at different fractions of transpirable soil water (FTSW). The FTSW represents the amount of water that can be extracted from soils to sustain whole-plant transpiration and ranges from 1 (maximum capacity of soil extraction) to 0 (plants can no longer extract water from soils to sustain transpiration). Breakpoints (BP) indicate the threshold FTSW at which transpiration starts to decline. **(A)** Contrasting sensitivity to soil dehydration between 2 maize hybrids. The maize hybrid 17 has higher sensitivity to soil dehydration than the hybrid 2, as demonstrated by the higher BP. Figure redrawn from Gholipoor et al. (2013) with permission from © 2013 John Wiley & Sons, Inc. **(B)** Contrasting sensitivity to soil dehydration of maize plants cultivated with 2 phosphorus fertilizers. Maize plants cultivated with triple super phosphate have a higher sensitivity to soil dehydration than plants cultivated with diammonium phosphate, as demonstrated by the higher BP. Figure redrawn from Sinclair et al. (2024) with permission from © 2024 Taylor & Francis, Inc.

Different crops and genotypes initiate stomatal closure at different points during a drought, ranging between 2 extremes of a spectrum (Fig. 3A) (Bartlett et al. 2014; Moshelion et al. 2015; Sinclair et al. 2018). “Water-saving” genotypes are highly sensitive to soil dehydration and close stomata early during drought, aiming to limit tissue dehydration and conserve soil water (Carminati et al. 2020). This behavior can be advantageous in sites where most rainfall occurs early in the growing season and terminal droughts are a threat (Zaman-Allah et al. 2011; Blum 2015). “Water-spending” genotypes are less sensitive to soil drying, delaying stomatal closure and thereby maintaining photosynthesis for a longer period during drought. In some cases, these genotypes exhibit heritable osmotic adjustment that supports the continued function at lower water potentials (Blum 2015).

The timing for stomatal closure during soil drought seems to be determined by a combination of plant traits and environmental factors (Koehler et al. 2023). Currently, 2 theories are used to explain stomatal closure of crops during drought. One postulates that during soil dehydration, leaf cells progressively decline in volume (Sack et al. 2018) and turgor (McAdam and Brodribb 2016), which triggers a gradual accumulation of foliar ABA—a hormone long associated with stomatal closure (Mittelheuser and Van Steveninck 1969; Beardsell and Cohen 1975). As soils and leaves continue to dehydrate, leaves completely lose turgor, experiencing a pronounced rise in ABA levels (McAdam and Brodribb 2016; Cardoso et al. 2020b) that results in maximum (>90%) stomatal closure. Another theory describes stomatal closure during drought as a function of declines in the soil-plant hydraulic conductances (Carminati and Javaux 2020; Wankmüller and Carminati 2022).

Among the plant traits influencing stomatal sensitivity to soil dehydration, we discuss root and leaf hydraulics. In root hydraulics, smaller effective root systems or roots that rapidly lose physical contact with the soil tend to exhibit greater stomatal sensitivity to soil drying (Carminati and Javaux 2020; Abdalla et al. 2022; Cai et al. 2022; Koehler et al. 2023). Regarding root hydraulic conductance (K_root_), 2 nonmutually exclusive pathways can produce earlier stomatal downregulation. First, when pre-drought K_root_ is low, faster plant dehydration occurs upon soil drying, leading to earlier ABA accumulation and stomatal closure (Sinclair et al. 2024). Second, when pre-drought K_root_ is high, tighter soil-plant coupling means that, as soils dry and soil hydraulic conductivity collapses, the soil side becomes limiting at a higher Ψ_soil_, steepening the soil-root interface gradient and likewise prompting earlier stomatal closure (Cai et al. 2022; Koehler et al. 2023). Which pathway dominates will depend on atmospheric demand, rhizosphere/substrate properties, rooting volume, and genotype. Irrespective of the maximum, pre-drought levels, K_root_ declines as soils dehydrate (via aquaporin regulation, loss of soil-root contact, and shrinking active root length), thereby contributing to stomatal closure during drought (Cai et al. 2022).

In addition to variations in maximum pre-drought K_root_ among species and genotypes, contrasting values of K_root_ have been found in maize plants cultivated with different phosphorus fertilizers, and plants with lower K_root_ exhibited greater stomatal sensitivity to soil dehydration (Fig. 3B) (Sinclair et al. 2024). The mechanisms through which specific phosphorus fertilizers affect K_root_ remain uncertain, yet this finding casts light on the exciting possibility of improving crop resistance to drought by manipulating root hydraulics via plant nutrition.

In leaf hydraulics, two traits are particularly important for the stomatal responses to soil dehydration: Ψ_TLP_ and declines in leaf hydraulic conductance (K_leaf_) during drought. Plants with a higher Ψ_TLP_ tend to be more sensitive to soil drying because turgor declines and foliar ABA accumulates at higher Ψ_leaf_ (McAdam and Brodribb 2016; Cardoso et al. 2020b; Corso et al. 2020). Variations in Ψ_TLP_ are typically narrow among crops and genotypes (<1 mPa) compared with variations among non-crops (Table 1) (Bartlett et al. 2014; Pereira et al. 2024). Still, with everything else equal, small differences in Ψ_TLP_ could potentially separate a productive from a nonproductive crop during drought. It is important to note that Ψ_TLP_ obtained in well-watered plants might not be representative of the Ψ_leaf_ at which plants will close their stomata during drought, especially for plants capable of osmotically adjusting. This might explain why some studies show stomatal closure at Ψ_leaf_ far more negative than the Ψ_TLP_ (Jacob et al. 2022).

**Table 1.** Mean values and standard error of water potentials at leaf turgor loss, 50% decline in leaf hydraulic conductance (k_leaf_), and 50% cumulative leaf embolism for 5 economically important crops

	Turgor loss point (MPa)	50% decline in K_leaf_ (MPa)	50% leaf embolism (MPa)
Sunflower	−0.80 ± 0.03	−0.53 ± 0.06	−1.86 ± 0.12
Tomato	−0.85 ± 0.02	−1.41 ± 0.10	−1.37 ± 0.06
Common bean	−0.80 ± 0.05	−0.91 ± 0.05	−1.41 ± 0.05
Cowpea	−0.85 ± 0.02	−0.61 ± 0.08	−1.36 ± 0.08
Soybean	−1.11 ± 0.08	−1.09 ± 0.007	−2.01 ± 0.07

Data were obtained by Moab and Cardoso (unpublished results).

Leaves decline K_leaf_ as soils and leaves dehydrate (Table 1). Reported declines have been attributed to impaired outside-xylem water transport (e.g. aquaporin deactivation) (Cochard et al. 2007; Scoffoni et al. 2017, 2023) and xylem embolism (Brodribb et al. 2016). In several species, including crop species and genotypes (Table 1), *g*_s_ and K_leaf_ decrease concomitantly during dehydration (Albuquerque et al. 2020; Corso et al. 2020), which amplifies stomatal closure. In other species and genotypes, K_leaf_ remains comparatively stable even as *g*_s_ falls (Bourbia and Brodribb 2024). Mechanistically, a lower K_leaf_ will, for a given transpiration, impose a larger water-potential drop within the leaf, lowering turgor and triggering ABA accumulation. In addition to roots and soils, leaf hydraulic efficiency and safety are central players contributing to stomatal closure during drought (Scoffoni et al. 2018).

### Increased stomatal sensitivity to VPD

In addition to soil drought, crops gradually close their stomata with rising VPD, preventing excessive water loss (Dai et al. 1992; Bunce 1997; Ohsumi et al. 2008; McAdam et al. 2016; Sinclair et al. 2017; Cardoso et al. 2018; Binstock et al. 2024) and avoiding severe leaf dehydration and damage (Brodribb et al. 2021). However, that does not necessarily mean that transpiration drops with rising VPD. In fact, transpiration typically increases with rising VPD despite partial stomatal closure because of the greater evaporative demand (Supplementary Fig. S1) (Dai et al. 1992; Bunce 1997). Note that leaf transpiration rate = stomatal conductance × VPD/atmospheric pressure (Martins et al. 2016). A possible disadvantage of stomatal closure under high VPD to plants is the limitation to photosynthesis, carbon gain, and yield. However, simulations show that declines in leaf gas exchange around midday when VPD is high have no or low penalty on yield in well-watered environments. Simulations include sorghum (Sinclair et al. 2005) and wheat (Collins et al. 2021) grown in Australia and soybean (Sinclair et al. 2010) and maize (Messina et al. 2015) grown in the US. The same studies show consistent increases in yield for all crops in water-limited environments due to soil water conservation.

**Figure 4.**
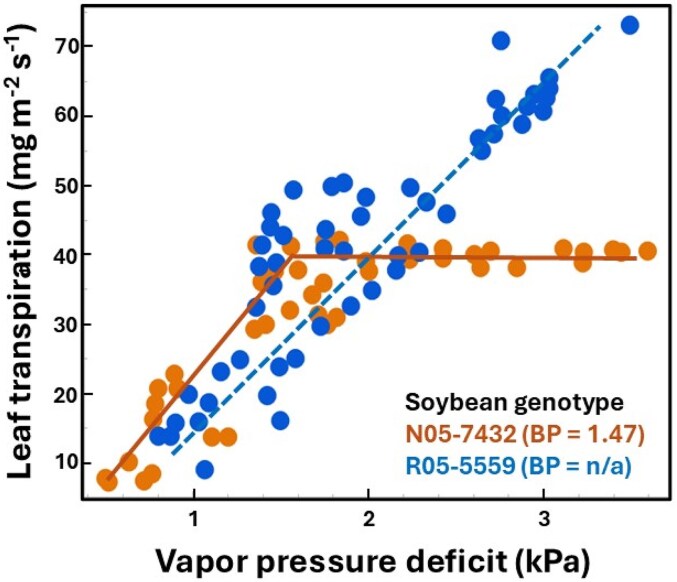
Contrasting stomatal sensitivity to VPD demonstrated by whole-plant transpiration responses. The soybean genotype N05-7432 has a higher stomatal sensitivity to VPD than the genotype R05-5559, which is demonstrated by its inability to maintain a linear increase in transpiration with rising VPD. The VPD threshold (breakpoint, BP) at which N05-7432 stops increasing transpiration linearly with rising VPD is 1.47 kPa. Figure redrawn from Devi et al. (2014) with permission from © 2014 John Wiley & Sons, Inc.

In well-watered soils, the ability of plants to limit increases in transpiration with rising VPD varies among crops and genotypes and directly reflects their stomatal sensitivity to evaporative demand (Sinclair et al. 2017; Koehler et al. 2023). Two contrasting responses of leaf transpiration to VPD are observed (Fig. 4): a linear increase in transpiration with rising VPD and a segmented response characterized by an initial linear increase until a VPD threshold (breakpoints ranging from 1.3 to 3.03 kPa) followed by a second linear regression showing low to no increases in transpiration with VPD (Sinclair et al. 2017; Koehler et al. 2023). Plants exhibiting a segmented response with transpiration stability at lower VPD thresholds are considered to have higher sensitivity to VPD, as stability is driven by stomatal closure (Koehler et al. 2023). Alternatively, Oren et al. (1999) described the stomatal sensitivity to VPD as the slope between *g*_s_ and ln(VPD). The former method is widely used by agronomists and crop physiologists, and the latter is commonly used by ecologists and ecophysiologists.

In crops, stomatal closure to VPD is thought to be mediated by foliar ABA (McAdam et al. 2016; Cardoso et al. 2020b; Brodribb et al. 2021; Binstock et al. 2024). A recent study using a herbaceous species (*Senecio minimus*) demonstrated that stomatal closure to VPD only occurs when VPD is high enough to cause transient declines in leaf cell turgor and trigger ABA accumulation (Binstock et al. 2024). According to the mechanism proposed by this study, it is intuitive to hypothesize that leaves with lower maximum K_leaf_ would fail to replace the water lost via transpiration under high VPD, thus experiencing rapid reductions in cell volume/turgor and ultimately resulting in ABA accumulation and stomatal closure at comparatively lower VPDs. Accordingly, differences between 2 soybean genotypes with contrasting stomatal sensitivities to VPD have been attributed to differences in K_leaf_ (Sinclair et al. 2008). The soybean genotype PI 416937, which has a lower K_leaf_, has higher stomatal sensitivity to VPD than 2 other genotypes (Biloxi and A5959) with greater K_leaf_ (Sinclair et al. 2008). Similarly, the sorghum genotype SC15 with lower K_leaf_ exhibited greater restriction of leaf transpiration to rising VPD than the genotype SC1205 with higher K_leaf_ (Choudhary et al. 2013). Lower K_root_ is also associated with increased stomatal sensitivity to VPD. The wheat genotype RAC875 has lower K_root_ and greater stomatal sensitivity to VPD than the genotype Kukri (Schoppach et al. 2014). Finally, a recent study used a simulation to confirm that lower plant hydraulic conductance results in higher stomatal sensitivity to VPD (Koehler et al. 2023). Therefore, low hydraulic conductance genotypes might outcompete genotypes with high hydraulic conductances in water-limited environments due to a higher sensitivity to VPD. It remains to be determined whether leaves or roots exert a greater influence on stomatal sensitivity to VPD.

A relatively unexplored player in this topic is leaf capacitance (C_leaf_), defined as the change in water content for a given change of Ψ_leaf_ (Nadal et al. 2023). The most immediate role of C_leaf_ is to protect plant tissues from large short-term drops in Ψ_leaf_ that could occur during a transition from low to high VPD, for example. Both K_leaf_ and C_leaf_ determine how fast a change in Ψ_leaf_ will occur upon a change in transpiration since the time constant is given by the ratio of C_leaf_ over K_leaf_ (Martins et al. 2016). In the present context, it seems reasonable to assume that a low C_leaf_ would contribute to a higher sensitivity to VPD, but the ratio of C_leaf_ over K_leaf_ in genotypes with contrasting responses to VPD remains to be tested.

### Decreased stomatal conductance

**Figure 5.**
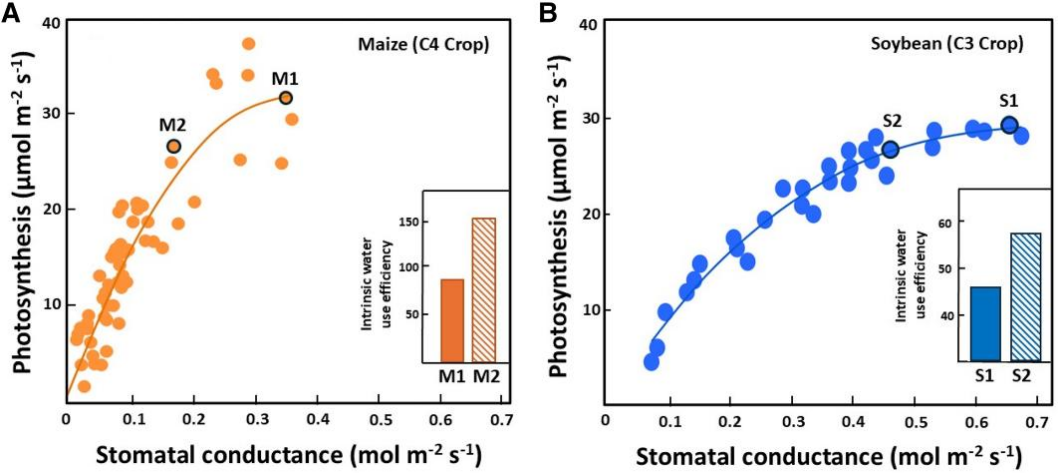
Relationships between stomatal conductance and photosynthesis follow a non-linear regression. **(A, B)** In C3 (soybean) and C4 (maize) crops, the relationship between stomatal conductance and photosynthesis follows a nonlinear regression. Increases in intrinsic WUE (photosynthesis/stomatal conductance) with low or no declines in photosynthesis occur as operational stomatal conductance declines from Supplementary S1 to S2 in soybean and M1 to M2 in maize. The soybean figure was redrawn from Gilbert et al. (2011) with permission from © 2011 Society for Experimental Biology. The maize figure was redrawn from Gleason et al. (2017) with permission from © 2017 Elsevier B.V.

In both C3 and C4 crops, the relationship between *g*_s_ and photosynthesis is nonlinear (Fig. 5). Initial increases in *g*_s_ cause pronounced gains in photosynthesis, but above a threshold, only marginal (if any) increases in photosynthesis occur due to nonstomatal limitations (Wong et al. 1979; Farquhar and Sharkey 1982). Under well-watered conditions, increases in intrinsic WUE (WUE_INT_ = net photosynthesis/*g*_s_) can be achieved via increases in photosynthetic capacity, reductions in *g*_s_, or both (Gilbert et al. 2011). Several studies, however, suggest that reducing *g*_s_ is more effective in enhancing WUE_INT_ than increasing photosynthesis (Blum 2005, 2009; Gilbert et al. 2011; Vadez et al. 2014). Moreover, lowering daytime *g*_s_ during well-watered conditions contributes to soil water conservation, thus reducing the probability of soil droughts developing (Vadez et al. 2014).

Recent investigations have employed genetic manipulations to reduce *g*_s_ and increase WUE in crops via reductions in stomatal density per unit leaf area (Hughes et al. 2017; Caine et al. 2019; Dunn et al. 2019; Li et al. 2020; Karavolias et al. 2023; Ferguson et al. 2024). These studies demonstrate that moderate reductions in stomatal density decrease *g*_s_ without significant decreases in photosynthesis or grain production. This is particularly true for C3 and C4 cereals (Hughes et al. 2017; Caine et al. 2019; Dunn et al. 2019; Karavolias et al. 2023; Ferguson et al. 2024). Reducing stomatal density and conductance at the leaf level was also demonstrated to decrease whole-plant water use and conserve soil water (Hughes et al. 2017; Caine et al. 2019; Ferguson et al. 2024). Future investigations should assess whether crops engineered to have reduced stomatal density (1) exhibit reduced nighttime transpiration (Box 2) and residual conductance (g_LEAF-RES_) (Box 3); (2) increase WUE while maintaining yield at the field level; (3) exhibit negative pleiotropic effects (unintended deleterious changes in other traits) that could be detrimental to plant development in the field (Ferguson et al. 2024); and (4) are suitable for crop production in water-limited environments. Constitutive increased levels of ABA have also been demonstrated to result in moderate decreases in *g*_s_ and increases in WUE without declines in photosynthesis (Thompson et al. 2007; Haverroth et al. 2023). The impacts of such increases in ABA levels for WUE at the plant level and productivity remain to be tested.

Box 2. Nighttime transpiration Plants lose water not only during the day when stomata are open and photosynthesis is occurring, but also at night due to incomplete stomatal closure (Caird et al. 2007; Howard and Donovan 2007; Sadok and Jagadish 2020; McAusland et al. 2021). Therefore, nighttime transpiration (*E*_night_) impacts both WUE and drought avoidance. Nighttime stomatal conductance is up to ten times higher than residual leaf conductance (Duursma et al. 2019), and the contribution of *E*_night_ to daily water loss can be considerable (from 5% to 50%) depending on the crop, genotype, and environment (Sadok and Jagadish 2020). As the globe experiences greater temperature increases during the night than during the day (Davy et al. 2017), the relevance of *E*_night_ might become even more apparent. Although typically costly for WUE, *E*_night_ may also confer context-dependent benefits, including release of respiratory CO₂, nutrient and/or oxygen uptake, overnight rehydration, and restoration of flow in partially embolized xylem, and leaf cooling on warm nights (Wang et al. 2021).

For C3 and C4 crops, *E*_night_ is decoupled from carbon gain and lowers plant-level WUE (Tolk et al. 2006; Hufstetler et al. 2007; Fish and Earl 2009; Coupel-Ledru et al. 2016; Sadok and Jagadish 2020). Besides reducing WUE, increased *E*_night_ depletes soil moisture and can threaten productivity under water limitation (Sadok and Jagadish 2020; López et al. 2021). Simple simulation models for wheat indicate that a 15% nighttime share of daily transpiration leads to 10% to 20% yield losses under water-limited conditions (López et al. 2021). Although our understanding of the mechanisms regulating stomatal responses at night is limited, recent work showed that nighttime transpiration declines with drought after species-specific soil-moisture thresholds, and its regulation can be hydropassive or ABA-mediated, with switches under severe stress; notably, an ABA-deficient tomato mutant failed to reduce night water loss (Fernández-de-Uña 2025; Pereira et al. 2025).

Variations in *E*_night_ are associated with changes in stomatal aperture throughout the night and in response to soil drought and VPD (Rawson and Clarke 1988; Caird et al. 2007; Chowdhury et al. 2022). Decreasing stomatal density may reduce nighttime conductance and water loss irrespective of the time of the night and the environmental condition (Coupel-Ledru et al. 2016); however, any strategy to lower nocturnal stomatal conductance should be tested case-by-case to confirm that the reduction is sufficient to decrease *E*_night_ and improve WUE without compromising the potential benefits highlighted above.

Box 3. Residual leaf transpiration At soil water potential (Ψ_soil_) lower than the PWP, leaves lose turgor, and transpiration continues at much lower rates defined by the leaf residual conductance (g_LEAF-RES_). The g_LEAF-RES,_ also known as minimum leaf conductance, represents the conductance of leaves occurring through the cuticle and stomata, when maximum stomatal closure has been achieved during drought (Duursma et al. 2019). Even after leaf turgor loss, some stomata remain open due to the presence of dust (Caird et al. 2007), malformation, and other physical limitations for closure (Duursma et al. 2019). Reduced g_LEAF-RES_ has long been considered an important drought avoidance trait in crops (Sinclair and Ludlow 1986; Muchow and Sinclair 1989), and recent studies further demonstrate that low g_LEAF-RES_ delays crop dehydration and xylem embolism accumulation (Sinclair 2000; Haverroth et al. 2023; Max et al. 2023; Pereira et al. 2024). Notably, g_LEAF-RES_ influences water loss under severe droughts in which the Ψ_soil_ is lower than the PWP, and the importance of g_LEAF-RES_ to crop production during drought remains to be demonstrated.

Wide variation in g_LEAF-RES_ has been demonstrated across crops (Duursma et al. 2019) and within genotypes of soybean (James et al. 2008), peanut (Rosas-Anderson et al. 2014), sorghum (Muchow and Sinclair 1989), rice (Saito and Futakuchi 2010), and wheat (Araus et al. 1991). In sorghum (Muchow and Sinclair 1989) and rice (Saito and Futakuchi 2010), genotypes with higher stomatal density exhibit higher g_LEAF-RES_, suggesting a significant role of incomplete stomatal closure to residual transpiration in these crops. In peanuts (Rosas-Anderson et al. 2014) and wheat (Araus et al. 1991), stomatal density did not associate with g_LEAF-RES_, demonstrating that other factors, such as the fraction of stomatal opening (the ability to efficiently close stomata) and cuticle conductance, contribute to g_LEAF-RES_ (Ochoa et al. 2024). Still, with everything else remaining the same (cuticle characteristics and the fraction of stomatal opening), genetic manipulations increasing WUE via reductions in stomatal density (Hughes et al. 2017; Caine et al. 2019; Dunn et al. 2019; Li et al. 2020; Ferguson et al. 2024) might also result in reduced g_LEAF-RES_, which should be confirmed in future studies.

## Hydraulic traits contributing to drought tolerance

Crops grown in water-limited environments can experience severe dehydration [Ψ_soil_ < PWP]. This is particularly true (1) early during the growing season when their young roots are shallow and limited to superficial soils that often experience lower water potentials; (2) during prolonged droughts (Subramanian et al. 1997); or (3) when soil droughts are accompanied by high temperatures and VPD (McAusland et al. 2023). About scenario (1), it is important to note that at early stages, plant leaf area is small and evapotranspiration is mostly associated with soil evaporation. During severe drought, plants might experience tissue and cell damage (Johnson et al. 2018; Cardoso et al. 2020a; Brodribb et al. 2021) and have impaired recovery from drought. Several authors consider that crops exposed to this level fail to yield at profitable levels. However, this might not be true for all crops and drought events. As global temperature and VPD rise due to climate change, crops are expected to experience increasingly severe droughts and dehydration levels (Scheff and Frierson 2015; Berg et al. 2017), and tolerance traits might become essential for future agriculture. Increased drought tolerance can also allow plants to exhibit lower stomatal sensitivity to drought, maintaining carbon assimilation and accumulation at more negative Ψ_soil_ without experiencing tissue damage; this can be seen as an alternative to limited transpiration traits (Gleason et al. 2025). The following paragraphs describe 2 drought tolerance mechanisms influenced by plant hydraulics that might benefit crops under certain environments.

### Osmotic adjustment

Osmotic adjustment refers to the plant's ability to accumulate solutes in tissues (leaves and roots), maintaining higher relative water content and turgor at lower Ψ_leaf_ (Blum 2005). The maintenance of cell turgor allows plants to sustain cell growth and stomatal opening, thus photosynthesizing longer during droughts (Blum 2017). Osmotic adjustment constitutes an important tolerance mechanism that allows plants to survive drought and salinity in natural environments (Bartlett et al. 2012, 2014). However, the contribution of osmotic adjustment for yield maintenance is debatable (Serraj and Sinclair 2002; Blum 2017; Turner 2018). By sustaining leaf gas exchange, osmotically adjusted plants continue to lose water through transpiration and depleting soil water (i.e. water-spender behavior). However, osmotic adjustment also maintains root cell turgor, allowing roots to grow deeper while maintaining a water potential gradient between plants and soils that ensures greater soil water extraction (Kusaka et al. 2005; Velázquez-Márquez et al. 2015). Therefore, the additional water lost due to osmotic adjustment can be seen as a long-term investment that can pay off if plants develop deeper roots capable of reaching different pools of soil water. Whether the carbon investment in osmotic adjustment results in a yield penalty is likely to depend on both species and environmental conditions.

In a recent review, Blum (2017) summarizes a series of studies correlating osmotic adjustment and yield during drought in several cereals and legumes. Still, studies can be found demonstrating that osmotic adjustment does not consistently increase yield in water-limited environments (Leport et al. 1999; Turner et al. 2006). Variations occur across genotypes, with osmotic adjustment increasing yield in some genotypes while decreasing in others. Variations also occur with drought intensity, with stronger associations between osmotic adjustment and yield under more severe droughts (Morgan 1983, 1984; Moinuddin et al. 2005). Last, a study in chickpea demonstrates that the phenotypic expression of osmotic adjustment might not be stable from year to year within the same genotype and that osmotic adjustment does not consistently increase yields under terminal droughts in this species (Turner et al. 2006).

Osmotic adjustment might be better suited for specific crops, given that not all crops and genotypes have the genetic capacity to osmotically adjust (Blum 2017), display stability in their osmotic adjustment over time (Turner et al. 2006), and increase yield in water-limited environments upon osmotic adjustment (Serraj and Sinclair 2002; Turner et al. 2006). For crops potentially benefiting from osmotic adjustment (e.g. wheat, Serraj and Sinclair 2002; Richards 2006), this mechanism seems more advantageous under longer and more severe droughts (Morgan 1983, 1984; Serraj and Sinclair 2002; Moinuddin et al. 2005), where terminal droughts are not common (Turner et al. 2006), and in environments with stored subsoil water, where developing deeper roots with superior capacity for extracting soil moisture pays off. Osmotic adjustment in combination with greater tolerance to dehydration-induced damage (Cardoso et al. 2018) might also be beneficial in environments experiencing intermittent droughts of mild to moderate intensity, in which plants can sustain photosynthesis and growth without incurring damage.

### Embolism resistance and stomatal safety margin

Xylem embolism occurs after a threshold water potential at which air enters the xylem conducting cells, rapidly expanding and forming an air cavity that blocks the water flow in that cell (Tyree and Sperry 1989). Xylem embolism reduces the ability of plants to transport water across organs and tissues, ultimately resulting in further dehydration. When occurring in leaves, xylem embolism results in necrosis (Johnson et al. 2018; Cardoso et al. 2020a; Brodribb et al. 2021) and shedding (Hochberg et al. 2017). When occurring in stems (particularly of woody plants), extensive embolism levels result in increasing mortality risk of whole plants (Choat et al. 2018; Brodribb et al. 2020).

Surprisingly, the impact of xylem embolism on yield has never been examined, to our knowledge, and our understanding of this topic is limited to circumstantial evidence. For instance, one could try to understand the impact of leaf embolism and damage on yield through several studies assessing the impact of leaf defoliation on yield. Regarding the loss of leaf area only, in soybean, defoliation experiments consistently show that yield is not negatively impacted by reductions in leaf area up to 35% during vegetative stages (until flowering) and up to 20% during reproductive stages (from flowering to grain maturity) (Owen et al. 2013). In maize, complete leaf removal does not result in yield losses when occurring at or before the growth stage V4 (when plants have 4 leaves with visible leaf collars) (Pearson and Fletcher 2009). While these findings demonstrate that crops can potentially tolerate leaf area loss without a yield penalty, it is yet to be determined whether minor to moderate leaf area loss due to xylem embolism would occur at no additional yield cost. The complicating factor here is to disentangle the yield penalty caused by whole-plant dehydration to the point of triggering xylem embolism (particularly when considering an extended drought period) and the additional penalty caused by xylem embolism itself. For instance, when plants experience such a dehydration level as that to trigger embolism in leaves, other tissues and cells might be impaired by the same dehydration level (e.g. reduced growth, embolism in stems and roots, and accumulation of reactive oxygen species).

**Figure 6.**
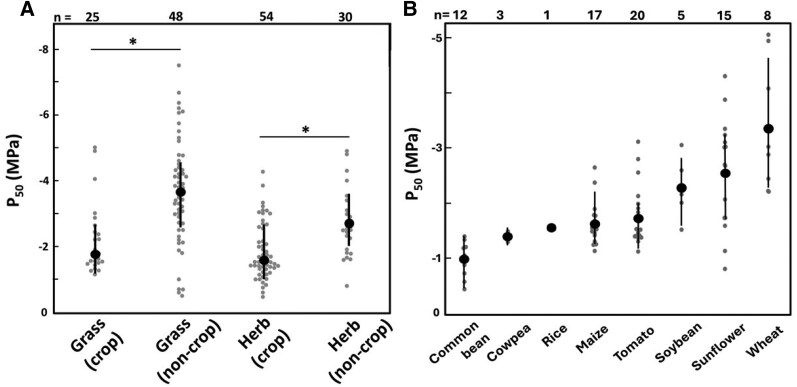
Resistance against hydraulic dysfunction in herbs and grasses. Gray dots are the original data. Black symbols are means ± standard error (*n* = top numbers). **(A)** Water potentials at 50% decline in hydraulic conductance or 50% embolism accumulation (P_50_) of grasses (crops and non-crops) and herbs (crops and non-crops). Asterisks denote statistical differences (Student's *t* test, *P* < 0.05 between crops and non-crops. **(B)** The P_50_ of crops (grasses and herbs) from the least to the most resistant. The P_50_ values were obtained from the Xylem Functional Traits Database and through a direct search on Google Scholar for new works involving the hydraulic vulnerability of crops with the keywords “Embolism vulnerability” + “Hydraulic vulnerability”, “Embolism vulnerability” + “Crop name”, “Xylem vulnerability” + “Crop name”. When the P_50_ data were not explicit, the corresponding author was contacted, or data were extracted using WebPlotDigitizer. Original data and references can be found in the Supplementary Data Set.

Given the absolute lack of data on the impact of xylem embolism on yield but the abundance of reports on the impact of embolism on crop function (Cardoso et al. 2018; Alves et al. 2024; Andrade et al. 2024; Pereira et al. 2024; Allen et al. 2025), it is reasonable to consider that crops avoiding xylem embolism during drought would perform and possibly yield better than those experiencing embolism (Gleason et al. 2022; Allen et al. 2025). For instance, based on the defoliation studies described above, leaf embolism and leaf area loss beyond a certain threshold are expected to reduce the plant's photosynthetic capacity, ultimately affecting yield. In stems, xylem embolism could not only prevent the transport of water but also prevent the remobilization of sugars from stems to grains, an important factor in grain filling during drought (Blum et al. 1997). Plants can prevent or at least minimize xylem embolism by exhibiting greater xylem resistance to embolism, slowing down tissue dehydration and the initiation of embolism formation, or both. Herbaceous crops, including eudicots and monocots, noticeably display less resistant xylem than non-crops (Fig. 6A). However, considerable variation in xylem resistance occurs across crop species (Fig. 6B) and genotypes (Supplementary Dataset) (Gleason et al. 2019; Matzner et al. 2019; Torres-Ruiz et al. 2024). Some crops have also been demonstrated to increase their xylem resistance as they mature (Haverroth et al. 2024) and upon drought exposure (Cardoso et al. 2018; Alves et al. 2024). Therefore, crops might exhibit natural genetic variation from which increased xylem resistance can be selected.

Even more important than increased xylem embolism resistance might be the combination of xylem resistance with early stomatal closure during drought and reduced g_LEAF-RES_ (Gleason et al. 2022; Pereira et al. 2024). Stomatal safety margin refers to the distance, in terms of Ψ_leaf_, between complete stomatal closure and the initiation of xylem embolism, which is associated with the time a plant can remain without soil water before experiencing embolism. Crops have been demonstrated to have very narrow safety margins (Torres-Ruiz et al. 2024), pushing plants to maintain leaf gas exchange and photosynthesis close to the onset of embolism formation. This trait might have been selected in conditions of optimum water supply, but it represents a risky strategy for crops under drought. Other plant traits influencing the time to embolism initiation (also referred to as the time for hydraulic failure) are g_LEAF-RES_ and plant capacitance (Blackman et al. 2019). Plants with similar stomatal safety margins will take longer to initiate embolism if they have reduced stomatal conductance and g_LEAF-RES_ as well as greater capacitance. Interestingly, the time for hydraulic failure combines drought tolerance (xylem embolism resistance) and drought avoidance traits (e.g. stomatal sensitivity to dehydration, g_LEAF-RES,_ and plant capacitance, among others), which might constitute an interesting phenotypic measure to select crops sustaining production in a future of severe droughts. The challenge is to assess the time for hydraulic failure in a high-throughput manner so that multiple genetic lines can be assessed for breeding programs.

Rather than emphasizing xylem resistance per se, we also highlight the underappreciated role of root pressure on xylem refilling and the recovery of hydraulic conductance in crops. In maize, genotypes with higher grain production under water limitation exhibited greater whole-shoot hydraulic conductivity and stronger pressurized root flow after rewatering—a functional readout that correlates with directly measured root pressure. This is consistent with positive xylem pressure recharging hydraulic capacitance and sustaining transpiration (Comas et al. 2025). In sorghum, root pressure can be induced upon rewatering from a drought, and its magnitude depends on the rewatering level and genotype. As it co-varies with the proportional investment in fine roots and shoot biomass, these patterns are compatible with a regulated, refilling-oriented function (Drobnitch et al. 2021). Future studies should quantify root pressure-driven refilling as an operational trait, its importance in stabilizing grain set under intermittent drought, and test its value as a selection criterion in breeding programs.

## Conclusions and future perspectives

Plant hydraulic traits are closely related to drought avoidance and tolerance in crops, which are critical for production under contrasting scenarios. Limited transpiration traits, critical for drought avoidance and yield stability under terminal droughts, are influenced by plant hydraulic conductances (controlling water supply) and stomatal structure and aperture (controlling water demand). Besides identifying genotypes with greater drought avoidance, manipulating high-yielding genotypes to minimize water use via genetic tools (Gray and Dunn 2024) and plant nutrition (Sinclair et al. 2024) emerge as novel approaches to improve drought avoidance. Osmotic adjustment is a drought tolerance mechanisms resulting in greater yield under severe droughts. Preventing xylem embolism and/or refiling embolized xylem upon rehydration represent drought tolerance mechanisms critical for plant survival during severe drought, but their contribution to crop production during drought is yet to be demonstrated. Time for hydraulic failure combines drought avoidance and tolerance, and its importance for crop production during moderate and severe droughts should be assessed.

Advances box

- Plant hydraulics and stomatal traits directly influence the ability of crops to grow and produce under water-limited conditions.
- Limited transpiration traits result in greater WUE and contribute to crop production during moderate and terminal droughts.
- Manipulating crops to reduce hydraulic and stomatal conductances appears to be an interesting approach to improve drought avoidance.
- Osmotic adjustment stands as an important tolerance mechanism that improves crop production during severe droughts.

Outstanding questions box

- Are stomatal sensitivity to soil dehydration and VPD influenced more by root or leaf hydraulic conductances? To what extent do leaf hydraulic conductance and capacitance influence the stomatal sensitivity to VPD?
- Do crops engineered to have reduced stomatal density exhibit lower nighttime and residual leaf conductances? Will crops engineered to have reduced stomatal density increase water use while maintaining yield at the field level? Do they represent an effective alternative to reduce irrigation needs and/or maintain crop production during drought?
- Do crops engineered to have increased levels of abscisic acid maintain satisfactory yields? Do they represent a viable alternative to reduce irrigation needs and/or maintain crop production during drought?
- Which nutrients and fertilizers most effectively modulate root hydraulics across crops and soils?
- Can crop production benefit from increased drought tolerance upon increasingly severe droughts? What crops and environments can benefit the most from increased drought tolerance?
- What is the impact of xylem embolism on the yield of annual crops? How do plant capacitance and the capacity to refill stored water (e.g. via root pressure) scale in crops and sustain yield under intermittent droughts?
- Can genotypes with a high stomatal-to-plant hydraulic conductance ratio, combined with strong hydraulic capacitance and effective refilling, outperform limited-transpiration genotypes under intermittent droughts?
- Does time for hydraulic failure represent an interesting trait to determine the ability of crops and genotypes to maintain production during moderate and/or severe droughts?

## Supplementary Material

kiaf521_Supplementary_Data

## Data Availability

The data underlying this article will be shared on request to the corresponding author.

## Supplementary material

The following materials are available in the online version of this article.

**Supplementary Figure S1.** Gas exchange and WUE of cotton leaves with increases in VPD. (A) Concomitant increases in transpiration (closed circles) and decreases in stomatal conductance (open circles) with increases in VPD. (B) Contrasting responses of leaf WUE (WUE_L_ = photosynthesis/transpiration) (closed circles) and intrinsic WUE (WUE_INT_ = photosynthesis/stomatal conductance) (open circles) with increases in VPD. Because of the opposite behavior of stomatal conductance and transpiration with increases in VPD (A), concomitant increases in WUE_INT_ and decreases in WUE_L_ are typically observed (B). Therefore, many authors normalize WUE_L_ to a common VPD or simply use WUE_INT_ when comparing the WUE among genotypes (Leakey et al. 2019). Data are means ± standard error (*n* = 5). Original data can be found in the Supplementary Data Set. Data were obtained by Andrade and Cardoso (unpublished results).

## Dive Curated Terms

The following phenotypic, genotypic, and functional terms are of significance to the work described in this paper:

drought resistance Planteome: TO:0000276

initial AmiGo: PO:0004011

TLP Gramene: turgor loss point
TLP Araport: turgor loss point

## References

[kiaf521-B1] Abdalla M, Ahmed MA, Cai G, Wankmüller F, Schwartz N, Litig O, Javaux M, Carminati A. Stomatal closure during water deficit is controlled by below-ground hydraulics. Ann Bot. 2022:129(2):161–170. 10.1093/aob/mcab141 PMC8796668 34871349

[kiaf521-B2] Ahmed MA, Passioura J, Carminati A. Hydraulic processes in roots and the rhizosphere pertinent to increasing yield of water-limited grain crops: a critical review. J Exp Bot. 2018:69(13):3255–3265. 10.1093/jxb/ery183 29767797

[kiaf521-B3] Albuquerque C, Scoffoni C, Brodersen CR, Buckley TN, Sack L, McElrone AJ. Coordinated decline of leaf hydraulic and stomatal conductances under drought is not linked to leaf xylem embolism for different grapevine cultivars. J Exp Bot. 2020:71(22):7286–7300. 10.1093/jxb/eraa392 33306796

[kiaf521-B4] Allen BS, Stewart JJ, Polutchko SK, Ocheltree TW, Gleason SM. Long-term in vivo observation of maize leaf Xylem embolism, transpiration and photosynthesis during drought and recovery. Plant Cell Environ. 2025:48(6):4114–4125. 10.1111/pce.15414 39901747 PMC12050386

[kiaf521-B5] Alves R, Menezes-Silva P, Loram-Lourenço L, Abreu I, Alencar K, Sousa L, Almeida S, Aun M, Silva M, Vasconcelos-Filho S, et al Exploring the coordinated hydraulic plasticity across organs in soybean plants exposed to drought cycles. Environ Exp Bot. 2024:226:105871. 10.1016/j.envexpbot.2024.105871

[kiaf521-B6] Andrade MT, Cardoso AA, Oliveira LA, Pereira TS, Haverroth EJ, Souza GA, DaMatta FM, Zsögön A, Martins SCV. Enhanced drought resistance in tomato via reduced auxin sensitivity: delayed dehydration and improved leaf resistance to embolism. Physiol Plant. 2024:176(3):e14408. 10.1111/ppl.14408

[kiaf521-B7] Araus JL, Febrero A, Vendrell P. Epidermal conductance in different parts of durum wheat grown under Mediterranean conditions: the role of epicuticular waxes and stomata. Plant Cell Environ. 1991:14(6):545–558. 10.1111/j.1365-3040.1991.tb01525.x

[kiaf521-B8] Bartlett MK, Scoffoni C, Sack L. The determinants of leaf turgor loss point and prediction of drought tolerance of species and biomes: a global meta-analysis. Ecol Lett. 2012:15(5):393–405. 10.1111/j.1461-0248.2012.01751.x 22435987

[kiaf521-B9] Bartlett MK, Zhang Y, Kreidler N, Sun S, Ardy R, Cao K, Sack L. Global analysis of plasticity in turgor loss point, a key drought tolerance trait. Ecol Lett. 2014:17(12):1580–1590. 10.1111/ele.12374 25327976

[kiaf521-B10] Beardsell MF, Cohen D. Relationships between leaf water Status, abscisic acid levels, and stomatal resistance in maize and Sorghum. Plant Physiol. 1975:56(2):207–212. 10.1104/pp.56.2.207 PMC541790 16659273

[kiaf521-B11] Berg A, Sheffield J, Milly PCD. Divergent surface and total soil moisture projections under global warming. Geophys Res Lett. 2017:44(1):236–244. 10.1002/2016GL071921

[kiaf521-B12] Berger J, Palta J, Vadez V. Review: an integrated framework for crop adaptation to dry environments: responses to transient and terminal drought. Plant Sci. 2016:253:58–67. 10.1016/j.plantsci.2016.09.007 27968997

[kiaf521-B13] Bhatnagar-Mathur P, Rao JS, Vadez V, Dumbala SR, Rathore A, Yamaguchi-Shinozaki K, Sharma KK. Transgenic peanut overexpressing the DREB1A transcription factor has higher yields under drought stress. Mol Breed. 2014:33(2):327–340. 10.1007/s11032-013-9952-7

[kiaf521-B14] Binstock BR, Manandhar A, McAdam SAM. Characterizing the breakpoint of stomatal response to vapor pressure deficit in an angiosperm. Plant Physiol. 2024:194(2):732–740. 10.1093/plphys/kiad560 37850913

[kiaf521-B15] Blackman CJ, Li X, Choat B, Rymer PD, De Kauwe MG, Duursma RA, Tissue DT, Medlyn BE. Desiccation time during drought is highly predictable across species of *Eucalyptus* from contrasting climates. New Phytol. 2019:224(2):632–643. 10.1111/nph.16042 31264226

[kiaf521-B16] Blum A . Drought resistance, water-use efficiency, and yield potential—are they compatible, dissonant, or mutually exclusive? Aust J Agric Res. 2005:56(11):1159. 10.1071/AR05069

[kiaf521-B17] Blum A . Effective use of water (EUW) and not water-use efficiency (WUE) is the target of crop yield improvement under drought stress. Field Crops Res. 2009:112(2-3):119–123. 10.1016/j.fcr.2009.03.009

[kiaf521-B18] Blum A . Drought resistance - is it really a complex trait? Functional Plant Biol. 2011:38(10):753–757. 10.1071/FP11101 32480932

[kiaf521-B19] Blum A . Towards a conceptual ABA ideotype in plant breeding for water limited environments. Funct Plant Biol. 2015:42(6):502–513. 10.1071/FP14334 32480696

[kiaf521-B20] Blum A . Osmotic adjustment is a prime drought stress adaptive engine in support of plant production. Plant Cell Environ. 2017:40(1):4–10. 10.1111/pce.12800 27417527

[kiaf521-B21] Blum A, Golan G, Mayer J, Sinmena B. The effect of dwarfing genes on sorghum grain filling from remobilized stem reserves, under stress. Field Crops Res. 1997:52(1-2):43–54. 10.1016/S0378-4290(96)03462-4

[kiaf521-B22] Bourbia L, Brodribb TJ. Stomatal response to VPD is not triggered by changes in soil–leaf hydraulic conductance in Arabidopsis or Callitris. New Phytol. 2024:242(2):444–452. 38396304 10.1111/nph.19607

[kiaf521-B23] Brodribb T, Brodersen CR, Carriqui M, Tonet V, Rodriguez Dominguez C, McAdam S. Linking xylem network failure with leaf tissue death. New Phytol. 2021:232(1):68–79. 10.1111/nph.17577 34164816

[kiaf521-B24] Brodribb TJ, Powers J, Cochard H, Choat B. Hanging by a thread? Forests and drought. Science. 2020:368(6488):261–266. 10.1126/science.aat7631 32299945

[kiaf521-B25] Brodribb TJ, Skelton RP, Mcadam SAM, Bienaimé D, Lucani CJ, Marmottant P. Visual quantification of embolism reveals leaf vulnerability to hydraulic failure. New Phytol. 2016:209(4):1403–1409. 10.1111/nph.13846 26742653

[kiaf521-B26] Bunce JA . Does transpiration control stomatal responses to water vapour pressure deficit? Plant Cell Environ. 1997:20(1):131–135. 10.1046/j.1365-3040.1997.d01-3.x

[kiaf521-B27] Cai G, Ahmed MA, Abdalla M, Carminati A. Root hydraulic phenotypes impacting water uptake in drying soils. Plant Cell Environ. 2022:45(3):650–663. 10.1111/pce.14259 35037263 PMC9303794

[kiaf521-B28] Caine RS, Yin X, Sloan J, Harrison EL, Mohammed U, Fulton T, Biswal AK, Dionora J, Chater CC, Coe RA, et al Rice with reduced stomatal density conserves water and has improved drought tolerance under future climate conditions. New Phytol. 2019:221(1):371–384. 10.1111/nph.15344 30043395 PMC6492113

[kiaf521-B29] Caird MA, Richards JH, Donovan LA. Nighttime stomatal conductance and transpiration in C3 and C4 plants. Plant Physiol. 2007:143(1):4–10. 10.1104/pp.106.092940 17210908 PMC1761996

[kiaf521-B30] Cardoso AA, Batz TA, McAdam SAM. Xylem embolism resistance determines leaf mortality during drought in *Persea americana*. Plant Physiol. 2020a:182(1):547–554. 10.1104/pp.19.00585 PMC6945834 31624082

[kiaf521-B31] Cardoso AA, Brodribb TJ, Lucani CJ, DaMatta FM, McAdam SAM. Coordinated plasticity maintains hydraulic safety in sunflower leaves. Plant Cell Environ. 2018:41(11):2567–2576. 10.1111/pce.13335 29748980

[kiaf521-B32] Cardoso AA, Gori A, Da-Silva CJ, Brunetti C. Abscisic acid biosynthesis and signaling in plants: key targets to improve water use efficiency and drought tolerance. Appl Sci. 2020b:10(18):6322. 10.3390/app10186322

[kiaf521-B33] Carminati A, Ahmed MA, Zarebanadkouki M, Cai G, Lovric G, Javaux M. Stomatal closure prevents the drop in soil water potential around roots. New Phytol. 2020:226(6):1541–1543. 10.1111/nph.16451 32077111

[kiaf521-B34] Carminati A, Javaux M. Soil rather than Xylem vulnerability controls stomatal response to drought. Trends Plant Sci. 2020:25(9):868–880. 10.1016/j.tplants.2020.04.003 32376085

[kiaf521-B35] Choat B, Brodribb TJ, Brodersen CR, Duursma RA, López R, Medlyn BE. Triggers of tree mortality under drought. Nature. 2018:558(7711):531–539. 10.1038/s41586-018-0240-x 29950621

[kiaf521-B36] Choudhary S, Sinclair TR, Prasad PVV. Hydraulic conductance of intact plants of two contrasting sorghum lines, SC15 and SC1205. Funct Plant Biol. 2013:40(7):730–738. 10.1071/FP12338 32481145

[kiaf521-B37] Chowdhury FI, Arteaga C, Alam MS, Alam I, Resco de Dios V. Drivers of nocturnal stomatal conductance in C3 and C4 plants. Sci Total Environ. 2022:814:151952. 10.1016/j.scitotenv.2021.151952 34843766

[kiaf521-B38] Cochard H, Venisse J-S, Barigah TS, Brunel N, Herbette S, Guilliot A, Tyree MT, Sakr S. Putative role of aquaporins in Variable hydraulic conductance of leaves in response to light. Plant Physiol. 2007:143(1):122–133. 10.1104/pp.106.090092 17114274 PMC1761984

[kiaf521-B39] Collins B, Chapman S, Hammer G, Chenu K. Limiting transpiration rate in high evaporative demand conditions to improve Australian wheat productivity. In Silico Plants. 2021:3(1):diab006. 10.1093/insilicoplants/diab006

[kiaf521-B40] Comas LH, Gleason SM, Drobnitch ST, Chintamanani S, Bensen R. Greater productivity under drought among *Zea mays* genotypes is linked to plant hydraulic strategies. Ann Bot. 2025:mcaf177. 10.1093/aob/mcaf177 Online ahead of print. 40819289 PMC12718040

[kiaf521-B41] Corso D, Delzon S, Lamarque LJ, Cochard H, Torres-Ruiz JM, King A, Brodribb T. Neither xylem collapse, cavitation, or changing leaf conductance drive stomatal closure in wheat. Plant Cell Environ. 2020:43(4):854–865. 10.1111/pce.13722 31953855

[kiaf521-B42] Coupel-Ledru A, Lebon E, Christophe A, Gallo A, Gago P, Pantin F, Doligez A, Simonneau T. Reduced nighttime transpiration is a relevant breeding target for high water-use efficiency in grapevine. Proc Natl Acad Sci U S A. 2016:113(32):8963–8968. 10.1073/pnas.1600826113 PMC4987834 27457942

[kiaf521-B43] Dai Z, Edwards GE, Ku MSB. Control of photosynthesis and stomatal conductance in *Ricinus communis* L. (castor bean) by leaf to air vapor pressure deficit. Plant Physiol. 1992:99(4):1426–1434. 10.1104/pp.99.4.1426 PMC1080643 16669054

[kiaf521-B44] Davy R, Esau I, Chernokulsky A, Outten S, Zilitinkevich S. Diurnal asymmetry to the observed global warming. Int J Climatol. 2017:37(1):79–93. 10.1002/joc.4688

[kiaf521-B45] Delzon S . New insight into leaf drought tolerance. Funct Ecol. 2015:29(10):1247–1249. 10.1111/1365-2435.12500

[kiaf521-B46] Devi JM, Sinclair TR, Chen P, Carter TE. Evaluation of elite southern maturity soybean breeding lines for drought-tolerant traits. Agron J. 2014:106(6):1947–1954.

[kiaf521-B47] Drobnitch ST, Comas LH, Flynn N, Caballero I, Barton J, Wenz RW, Person J, Bushey T, Jahn J, Gleason CE, et al Drought-induced root pressure in *Sorghum bicolor*. Front Plant Sci. 2021:12:571072. 10.3389/fpls.2021.571072 33613594 PMC7886691

[kiaf521-B48] Dunn J, Hunt L, Afsharinafar M, Al Meselmani M, Mitchell A, Howells R, Wallington E, Fleming AJ, Gray JE. Reduced stomatal density in bread wheat leads to increased water-use efficiency. J Exp Bot. 2019:70(18):4737–4748. 10.1093/jxb/erz248 31172183 PMC6760291

[kiaf521-B49] Duursma RA, Blackman CJ, Lopéz R, Martin-StPaul NK, Cochard H, Medlyn BE. On the minimum leaf conductance: its role in models of plant water use, and ecological and environmental controls. New Phytol. 2019:221(2):693–705. 10.1111/nph.15395 30144393

[kiaf521-B50] Farquhar GD, Sharkey TD. Stomatal conductance and photosynthesis. Ann Rev Plant Bio. 1982:33(1):317–345. 10.1146/annurev.pp.33.060182.001533

[kiaf521-B51] Ferguson JN, Schmuker P, Dmitrieva A, Quach T, Zhang T, Ge Z, Nersesian N, Sato SJ, Clemente TE, Leakey ADB. Reducing stomatal density by expression of a synthetic epidermal patterning factor increases leaf intrinsic water use efficiency and reduces plant water use in a C4 crop. J Exp Bot. 2024:75(21):6823–6836. 10.1093/jxb/erae289 39021331 PMC11565208

[kiaf521-B52] Fernández-de-Uña L . Disentangling the mechanisms regulating nighttime transpiration during drought across plant life forms. Plant Physiol. 2025:198(4):kiaf331. 10.1093/plphys/kiaf331 40718997 PMC12360925

[kiaf521-B53] Fish DA, Earl HJ. Water-use efficiency is negatively correlated with leaf epidermal conductance in cotton (*Gossypium* spp.). Crop Sci. 2009:49(4):1409–1415. 10.2135/cropsci2008.08.0490

[kiaf521-B54] Gholipoor M, Sinclair TR, Raza MAS, Löffler C, Cooper M, Messina CD. Maize hybrid variability for transpiration decrease with progressive soil drying. J Agron Crop Sci. 2013:199(1):23–29.

[kiaf521-B55] Gilbert ME, Zwieniecki MA, Holbrook NM. Independent variation in photosynthetic capacity and stomatal conductance leads to differences in intrinsic water use efficiency in 11 soybean genotypes before and during mild drought. J Exp Bot. 2011:62(8):2875–2887. 10.1093/jxb/erq461 21339387

[kiaf521-B56] Gleason SM, Barnard DM, Green TR, Mackay S, Wang DR, Ainsworth EA, Altenhofen J, Brodribb TJ, Cochard H, Comas LH, et al Physiological trait networks enhance understanding of crop growth and water use in contrasting environments. Plant Cell Environ. 2022:45(9):2554–2572. 10.1111/pce.14382 35735161

[kiaf521-B57] Gleason SM, Cooper M, Wiggans DR, Bliss CA, Romay MC, Gore MA, Mickelbart MV, Topp CN, Zhang H, DeJonge KC, et al Stomatal conductance, xylem water transport, and root traits underpin improved performance under drought and well-watered conditions across a diverse panel of maize inbred lines. Field Crops Res. 2019:234:119–128. 10.1016/j.fcr.2019.02.001

[kiaf521-B58] Gleason SM, Polutchko SK, Allen BS, Ocheltree TW, Spitzer D, Li Z, Stewart JJ. A 50-year look-back on the efficacy of limited transpiration traits: does the evidence support the recent surge in interest? New Phytol. 2025:246(4):1439–1450. 10.1111/nph.70071 40156228

[kiaf521-B59] Gleason SM, Wiggans DR, Bliss CA, Comas LH, Cooper M, DeJonge KC, Young JS, Zhang H. Coordinated decline in photosynthesis and hydraulic conductance during drought stress in Zea mays. Flora. 2017:227:1–9.

[kiaf521-B60] Gray J, Dunn J. Optimizing crop plant stomatal density to mitigate and adapt to climate change. Cold Spring Harb Perspect Biol. 2024:16(6):a041672. 10.1101/cshperspect.a041672 37923396 PMC11146307

[kiaf521-B61] Grossiord C, Buckley TN, Cernusak LA, Novick KA, Poulter B, Siegwolf RTW, Sperry JS, McDowell NG. Plant responses to rising vapor pressure deficit. New Phytol. 2020:226(6):1550–1566. 10.1111/nph.16485 32064613

[kiaf521-B62] Haverroth EJ, Oliveira LA, Andrade MT, Taggart M, McAdam SAM, Zsögön A, Thompson AJ, Martins SCV, Cardoso AA. Abscisic acid acts essentially on stomata, not on the xylem, to improve drought resistance in tomato. Plant Cell Environ. 2023:46(11):3229–3241. 10.1111/pce.14676 37526514

[kiaf521-B63] Haverroth EJ, Rimer IM, Oliveira LA, de Lima LGA, Cesarino I, Martins SCV, McAdam SAM, Cardoso AA. Gradients in embolism resistance within stems driven by secondary growth in herbs. Plant Cell Environ. 2024:47(8):2986–2998. 10.1111/pce.14921 38644584

[kiaf521-B64] Hochberg U, Windt CW, Ponomarenko A, Zhang Y-J, Gersony J, Rockwell FE, Holbrook NM. Stomatal closure, basal leaf embolism, and shedding protect the hydraulic integrity of grape stems. Plant Physiol. 2017:174(2):764–775. 10.1104/pp.16.01816 28351909 PMC5462014

[kiaf521-B65] Howard AR, Donovan LA. Helianthus nighttime conductance and transpiration respond to soil water but not nutrient availability. Plant Physiol. 2007:143(1):145–155. 10.1104/pp.106.089383 PMC1761982 17142487

[kiaf521-B66] Hufstetler EV, Boerma HR, Carter TE, Earl HJ. Genotypic variation for three physiological traits affecting drought tolerance in soybean. Crop Sci. 2007:47(1):25–35. 10.2135/cropsci2006.04.0243

[kiaf521-B67] Hughes J, Hepworth C, Dutton C, Dunn JA, Hunt L, Stephens J, Waugh R, Cameron DD, Gray JE. Reducing stomatal density in barley improves drought tolerance without impacting on yield. Plant Physiol. 2017:174(2):776–787. 10.1104/pp.16.01844 PMC5462017 28461401

[kiaf521-B68] Jacob V, Choat B, Churchill AC, Zhang H, Barton CVM, Krishnananthaselvan A, Post AK, Power SA, Medlyn BE, Tissue DT. High safety margins to drought-induced hydraulic failure found in five pasture grasses. Plant Cell Environ. 2022:45(6):1631–1646. 10.1111/pce.14318 35319101

[kiaf521-B69] James AT, Lawn RJ, Cooper M. Genotypic variation for drought stress response traits in soybean. I. Variation in soybean and wild *Glycine* spp. For epidermal conductance, osmotic potential, and relative water content. Aust J Agric Res. 2008:59(7):656–669. 10.1071/AR07159

[kiaf521-B70] Johnson KM, Jordan GJ, Brodribb TJ. Wheat leaves embolized by water stress do not recover function upon rewatering. Plant Cell Environ. 2018:41(11):2704–2714. 10.1111/pce.13397 29981153

[kiaf521-B71] Karavolias NG, Patel-Tupper D, Seong K, Tjahjadi M, Gueorguieva G-A, Tanaka J, Gallegos Cruz A, Lieberman S, Litvak L, Dahlbeck D, et al Paralog editing tunes rice stomatal density to maintain photosynthesis and improve drought tolerance. Plant Physiol. 2023:192(2):1168–1182. 10.1093/plphys/kiad183 36960567 PMC10231365

[kiaf521-B72] Kimm H, Guan K, Gentine P, Wu J, Bernacchi CJ, Sulman BN, Griffis TJ, Lin C. Redefining droughts for the U.S. Corn belt: the dominant role of atmospheric vapor pressure deficit over soil moisture in regulating stomatal behavior of maize and soybean. Agric For Meteorol. 2020:287:107930. 10.1016/j.agrformet.2020.107930

[kiaf521-B73] Koehler T, Moser DS, Botezatu Á, Murugesan T, Kaliamoorthy S, Zarebanadkouki M, Bienert MD, Bienert GP, Carminati A, Kholová J, et al Going underground: soil hydraulic properties impacting maize responsiveness to water deficit. Plant Soil. 2022:478(1–2):43–58. 10.1007/s11104-022-05656-2

[kiaf521-B74] Koehler T, Wankmüller FJP, Sadok W, Carminati A. Transpiration response to soil drying versus increasing vapor pressure deficit in crops: physical and physiological mechanisms and key plant traits. J Exp Bot. 2023:74(16):4789–4807. 10.1093/jxb/erad221 37354081 PMC10474596

[kiaf521-B75] Kusaka M, Lalusin AG, Fujimura T. The maintenance of growth and turgor in pearl millet (Pennisetum glaucum [L.] leeke) cultivars with different root structures and osmo-regulation under drought stress. Plant Sci. 2005:168(1):1–14. 10.1016/j.plantsci.2004.06.021

[kiaf521-B76] Leakey ADB, Ferguson JN, Pignon CP, Wu A, Jin Z, Hammer GL, Lobell DB. Water use efficiency as a constraint and target for improving the resilience and productivity of C_3_ and C_4_ crops. Annu Rev Plant Biol. 2019:70(1):781–808. 10.1146/annurev-arplant-042817-040305 31035829

[kiaf521-B77] Leng G, Hall J. Crop yield sensitivity of global major agricultural countries to droughts and the projected changes in the future. Sci Tot Environ. 2019:654:811–821. 10.1016/j.scitotenv.2018.10.434 PMC6341212 30448671

[kiaf521-B78] Leport L, Turner NC, French RJ, Barr MD, Duda R, Davies SL, Tennant D, Siddique KHM. Physiological responses of chickpea genotypes to terminal drought in a Mediterranean-type environment. Eur J Agron. 1999:11(3–4):279–291. 10.1016/S1161-0301(99)00039-8

[kiaf521-B79] Levitt J . Responses of plants to environmental stresses. New York: Academic Press; 1980.

[kiaf521-B80] Li B, Zhang X, Morita S, Sekiya N, Araki H, Gu H, Han J, Lu Y, Liu X. Are crop deep roots always beneficial for combating drought: a review of root structure and function, regulation and phenotyping. Agric Water Manag. 2022:271:107781. 10.1016/j.agwat.2022.107781

[kiaf521-B81] Li G, Zhao J, Qin B, Yin Y, An W, Mu Z, Cao Y. ABA mediates development-dependent anthocyanin biosynthesis and fruit coloration in Lycium plants. BMC Plant Biol. 2019:19(1):1–13. 10.1186/s12870-019-1931-7 PMC6631627 31307384

[kiaf521-B82] Li S, Zhang J, Liu L, Wang Z, Li Y, Guo L, Li Y, Zhang X, Ren S, Zhao B, et al *SlTLFP8* reduces water loss to improve water-use efficiency by modulating cell size and stomatal density via endoreduplication. Plant Cell Environ. 2020:43(11):2666–2679. 10.1111/pce.13867 32799324

[kiaf521-B83] Liu J, Huang J, Peng S, Xiong D. Rewatering after drought: unravelling the drought thresholds and function recovery-limiting factors in maize leaves. Plant Cell Environ. 2024:47(12):5457–5469. 10.1111/pce.15080 39205650

[kiaf521-B84] Lobell DB, Roberts MJ, Schlenker W, Braun N, Little BB, Rejesus RM, Hammer GL. Greater sensitivity to drought accompanies maize yield increase in the U.S. Midwest. Science. 2014:344:516–519. 10.1126/science.1251423 24786079

[kiaf521-B85] López JR, Schoppach R, Sadok W. Harnessing nighttime transpiration dynamics for drought tolerance in grasses. Plant Signal Behav. 2021:16(4):1875646. 10.1080/15592324.2021.1875646 PMC7971256 33465000

[kiaf521-B86] Manschadi AM, Christopher J, deVoil P, Hammer GL. The role of root architectural traits in adaptation of wheat to water-limited environments. Funct Plant Biol. 2006:33(9):823–837. 10.1071/FP06055 32689293

[kiaf521-B87] Martins SCV, McAdam SAM, Deans RM, DaMatta FM, Brodribb TJ. Stomatal dynamics are limited by leaf hydraulics in ferns and conifers: results from simultaneous measurements of liquid and vapour fluxes in leaves. Plant Cell Environ. 2016:39(3):694–705. 10.1111/pce.12668 26510650

[kiaf521-B88] Matzner SL, Ronning N, Hawkinson J, Cummiskey T, Buchanan J, Miller E, Carlisle G. Does acclimation in cavitation resistance due to mechanical perturbation support the pit area or conduit reinforcement hypotheses in *Phaseolus vulgaris* ? Physiol Plant. 2019:167(3):378–390. 10.1111/ppl.12895 30537192 PMC6557702

[kiaf521-B89] Maurel C, Nacry P. Root architecture and hydraulics converge for acclimation to changing water availability. Nat Plants. 2020:6(7):744–749. 10.1038/s41477-020-0684-5 32601421

[kiaf521-B90] Max AC, Loram-Lourenço L, Silva FG, de Souza LHM, Dias JRM, Espíndula MC, Farnese FS, Hammond W, Torres-Ruiz JM, Cochard H, et al A bitter future for coffee production? Physiological traits associated with yield reveal high vulnerability to hydraulic failure in *Coffea canephora*. Plant Cell Environ. 2023:46(3):764–779. 10.1111/pce.14514 36517464

[kiaf521-B91] McAdam SAM, Brodribb TJ. Linking turgor with ABA biosynthesis: implications for stomatal responses to vapor pressure deficit across land plants. Plant Physiol. 2016:171(3):2008–2016. 10.1104/pp.16.00380 27208264 PMC4936570

[kiaf521-B92] McAdam SAM, Sussmilch FC, Brodribb TJ. Stomatal responses to vapour pressure deficit are regulated by high speed gene expression in angiosperms. Plant Cell Environ. 2016:39(3):485–491. 10.1111/pce.12633 26353082

[kiaf521-B93] McAusland L, Acevedo-Siaca LG, Pinto RS, Pinto F, Molero G, Garatuza-Payan J, Reynolds MP, Murchie EH, Yepez EA. Night-time warming in the field reduces nocturnal stomatal conductance and grain yield but does not alter daytime physiological responses. New Phytol. 2023:239(5):1622–1636. 10.1111/nph.19075 PMC10952344 37430457

[kiaf521-B94] McAusland L, Smith KE, Williams A, Molero G, Murchie EH. Nocturnal stomatal conductance in wheat is growth-stage specific and shows genotypic variation. New Phytol. 2021:232(1):162–175. 10.1111/nph.17563 34143507

[kiaf521-B95] Messina CD, Sinclair TR, Hammer GL, Curan D, Thompson J, Oler Z, Gho C, Cooper M. Limited-transpiration trait may increase maize drought tolerance in the US corn belt. Agron J. 2015:107(6):1978–1986. 10.2134/agronj15.0016

[kiaf521-B96] Mittelheuser CJ, Van Steveninck RFM. Stomatal closure and inhibition of transpiration induced by (RS)-abscisic acid. Nature. 1969:221(5177):281–282. 10.1038/221281a0

[kiaf521-B97] Moinuddin , Fischer RA, Sayre KD, Reynolds MP. Osmotic adjustment in wheat in relation to grain yield under water deficit environments. Agron J. 2005:97(4):1062–1071. 10.2134/agronj2004.0152

[kiaf521-B98] Morgan J . Osmoregulation as a selection criterion for drought tolerance in wheat. Aust J Agric Res. 1983:34(6):607–614. 10.1071/AR9830607

[kiaf521-B99] Morgan JM . Osmoregulation and water stress in higher plants. Annu Rev Plant Physiol. 1984:35(1):299–319. 10.1146/annurev.pp.35.060184.001503

[kiaf521-B100] Moshelion M, Halperin O, Wllach R, Orem R, Way DA. Role of aquaporins in determining transpiration and photosynthesis in water-stressed plants: crop water-use efficiency, growth and yield. Plant Cell Environ. 2015:38(9):1785–1793. 10.1111/pce.12410 25039365

[kiaf521-B101] Muchow RC, Sinclair TR. Epidermal conductance, stomatal density and stomatal size among genotypes of *Sorghum bicolor* (L.) moench. Plant Cell Environ. 1989:12(4):425–431. 10.1111/j.1365-3040.1989.tb01958.x

[kiaf521-B102] Mueller ND, Gerber JS, Johnston M, Ray DK, Ramankutty N, Foley JA. Closing yield gaps through nutrient and water management. Nature. 2012:490(7419):254–257. 10.1038/nature11420 22932270

[kiaf521-B103] Nadal M, Clemente-Moreno MJ, Perera-Castro AV, Roig-Oliver M, Onoda Y, Gulías J, Flexas J. Incorporating pressure–volume traits into the leaf economics spectrum. Ecol Lett. 2023:26(4):549–562. 10.1111/ele.14176 36750322

[kiaf521-B104] Ochoa ME, Henry C, John GP, Medeiros CD, Pan R, Scoffoni C, Buckley TN, Sack L. Pinpointing the causal influences of stomatal anatomy and behavior on minimum, operational, and maximum leaf surface conductance. Plant Physiol. 2024:196(1):51–66. 10.1093/plphys/kiae292 38775665

[kiaf521-B105] Ohsumi A, Hamasaki A, Nakagawa H, Homma K, Horie T, Shiraiwa T. Response of leaf photosynthesis to vapor pressure difference in rice (*Oryza sativa* L) varieties in relation to stomatal and leaf internal conductance. Plant Prod Sci. 2008:11(2):184–191. 10.1626/pps.11.184

[kiaf521-B106] Oliveira LA, Cardoso AA, Andrade MT, Pereira TS, Araújo WL, Santos GA, Damatta FM, Martins SCV. Exploring leaf hydraulic traits to predict drought tolerance of *Eucalyptus* clones. Tree Physiol. 2022:42(9):1750–1761. 10.1093/treephys/tpac040 35388901

[kiaf521-B107] Oren R, Sperry JS, Katul GG, Pataki DE, Ewers BE, Phillips N, Schäfer KVR. Survey and synthesis of intra- and interspecific variation in stomatal sensitivity to vapour pressure deficit. Plant Cell Environ. 1999:22(12):1515–1526. 10.1046/j.1365-3040.1999.00513.x

[kiaf521-B108] Owen LN, Catchot AL, Musser FR, Gore J, Cook DC, Jackson R, Allen C. Impact of defoliation on yield of group IV soybeans in Mississippi. Crop Protection. 2013:54:206–212. 10.1016/j.cropro.2013.08.007

[kiaf521-B109] Passioura JB . Grain yield, harvest Index, and water use of wheat. J Aust Inst Agric Sci. 1977:43:117–120.

[kiaf521-B110] Pearson A, Fletcher AL. Effect of total defoliation on maize growth and yield. Agronomy NZ. 2009:39:1–6.

[kiaf521-B111] Pereira TS, Manandhar A, Cardoso AA, Martins SC, McAdam SA. Divergent pathways across vascular plant lineages drive reduced nighttime transpiration during drought. Plant Physiol. 2025:198(4):kiaf274. 10.1093/plphys/kiaf274 40557976

[kiaf521-B112] Pereira TS, Oliveira LA, Andrade MT, Haverroth EJ, Cardoso AA, Martins SCV. Linking water-use strategies with drought resistance across herbaceous crops. Physiol Plant. 2024:176(1):e14114. 10.1111/ppl.14114

[kiaf521-B113] Pimentel D, Cerasale D, Stanley RC, Perlman R, Newman EM, Brent LC, Mullan A, Chang DT-I. Annual vs. Perennial grain production. Agric Ecosyst Environ. 2012:161:1–9. 10.1016/j.agee.2012.05.025

[kiaf521-B114] Ratnakumar P, Vadez V, Nigam SN, Krishnamurthy L. Assessment of transpiration efficiency in peanut (*Arachis hypogaea* L.) under drought using a lysimetric system. Plant Biol. 2009:11(s1):124–130. 10.1111/j.1438-8677.2009.00260.x 19778376

[kiaf521-B115] Rawson H, Clarke J. Nocturnal transpiration in wheat. Funct Plant Biol. 1988:15(3):397–406. 10.1071/PP9880397

[kiaf521-B116] Ray JD, Sinclair TR. Stomatal closure of maize hybrids in response to drying soil. Crop Sci. 1997:37(3):803–807. 10.2135/cropsci1997.0011183X003700030018x

[kiaf521-B117] Richards RA . Physiological traits used in the breeding of new cultivars for water-scarce environments. Agric Water Manag. 2006:80(1–3):197–211. 10.1016/j.agwat.2005.07.013

[kiaf521-B118] Rosa L, Chiarelli DD, Rulli MC, Dell’Angelo J, D’Odorico P. Global agricultural economic water scarcity. Sci Adv. 2020:6(18):eaaz6031. 10.1126/sciadv.aaz6031 32494678 PMC7190309

[kiaf521-B119] Rosas-Anderson P, Shekoofa A, Sinclair TR, Balota M, Isleib TG, Tallury S, Rufty T. Genetic variation in peanut leaf maintenance and transpiration recovery from severe soil drying. Field Crops Res. 2014:158:65–72.

[kiaf521-B120] Sack L, John GP, Buckley TN. ABA accumulation in dehydrating leaves is associated with decline in cell volume, not turgor pressure. Plant Physiol. 2018:176(1):489–495. 10.1104/pp.17.01097 29061906 PMC5761807

[kiaf521-B121] Sadok W, Jagadish SVK. The hidden costs of nighttime warming on yields. Trends Plant Sci. 2020:25(7):644–651. 10.1016/j.tplants.2020.02.003 32526169

[kiaf521-B122] Saito K, Futakuchi K. Genotypic variation in epidermal conductance and its associated traits among *Oryza sativa* and *O. glaberrima* cultivars and their interspecific progenies. Crop Sci. 2010:50(1):227–234. 10.2135/cropsci2009.06.0284

[kiaf521-B123] Sánchez-Gómez D, Cervera MT, Escolano-Tercero MA, Vélez MD, de María N, Diaz L, Sánchez-Vioque R, Aranda I, Guevara MÁ. Drought escape can provide high grain yields under early drought in lentils. Theor Exp Plant Physiol. 2019:31(2):273–286. 10.1007/s40626-018-0136-z

[kiaf521-B124] Scheff J, Frierson DMW. Scaling potential evapotranspiration with greenhouse warming. J Clim. 2014:27(4):1539–1558. 10.1175/JCLI-D-13-00233.1

[kiaf521-B125] Scheff J, Frierson DMW. Terrestrial aridity and its response to greenhouse warming across CMIP5 climate models. J Clim. 2015:28(14):5583–5600. 10.1175/JCLI-D-14-00480.1

[kiaf521-B126] Schoppach R, Wauthelet D, Jeanguenin L, Sadok W. Conservative water use under high evaporative demand associated with smaller root metaxylem and limited trans-membrane water transport in wheat. Funct Plant Biol. 2014:41(3):257–269. 10.1071/FP13211 32480986

[kiaf521-B127] Scoffoni C, Albuquerque C, Brodersen CR, Townes SV, John GP, Bartlett MK, Buckley TN, McElrone AJ, Sack L. Outside-Xylem vulnerability, not Xylem embolism, controls leaf hydraulic decline during dehydration. Plant Physiol. 2017:173(2):1197–1210. 10.1104/pp.16.01643 PMC5291720 28049739

[kiaf521-B128] Scoffoni C, Albuquerque C, Buckley TN, Sack L. The dynamic multi-functionality of leaf water transport outside the xylem. New Phytol. 2023:239(6):2099–2107. 10.1111/nph.19069 37386735

[kiaf521-B129] Scoffoni C, Albuquerque C, Cochard H, Buckley TN, Fletcher LR, Caringella MA, Bartlett M, Brodersen CR, Jansen S, McElrone AJ, et al The causes of leaf hydraulic vulnerability and its influence on gas exchange in *Arabidopsis thaliana*. Plant Physiol. 2018:178(4):1584–1601. 10.1104/pp.18.00743 PMC6288733 30366978

[kiaf521-B130] Serraj R, Sinclair TR. Osmolyte accumulation: can it really help increase crop yield under drought conditions? Plant Cell Environ. 2002:25(2):333–341. 10.1046/j.1365-3040.2002.00754.x 11841674

[kiaf521-B131] Shavrukov Y, Kurishbayev A, Jatayev S, Shvidchenko V, Zotova L, Koekemoer F, de Groot S, Soole K, Langridge P. Early flowering as a drought Escape mechanism in plants: how can it aid wheat production? Front Plant Sci. 2017:8:1950. 10.3389/fpls.2017.01950 29204147 PMC5698779

[kiaf521-B132] Simpson E, Haverroth EJ, Taggart M, Andrade MT, Villegas DA, Carbajal EM, Oliveira LA, Suchoff D, Milla-Lewis S, Cardoso AA. Dehydration tolerance rather than avoidance explains drought resistance in zoysiagrass. Physiol Plant. 2024:176(6):e14622. 10.1111/ppl.14622 39557073

[kiaf521-B133] Sinclair TR . Model analysis of plant traits leading to prolonged crop survival during severe drought. Field Crops Res. 2000:68(3):211–217. 10.1016/S0378-4290(00)00125-8

[kiaf521-B134] Sinclair TR . Challenges in breeding for yield increase for drought. Trends Plant Sci. 2011:16(6):289–293. 10.1016/j.tplants.2011.02.008 21419688

[kiaf521-B135] Sinclair TR, Devi J, Shekoofa A, Choudhary S, Sadok W, Vadez V, Riar M, Rufty T. Limited-transpiration response to high vapor pressure deficit in crop species. Plant Sci. 2017:260:109–118. 10.1016/j.plantsci.2017.04.007 28554468

[kiaf521-B136] Sinclair TR, Hammer GL, Van Oosterom EJ. Potential yield and water-use efficiency benefits in sorghum from limited maximum transpiration rate. Funct Plant Biol. 2005:32(10):945–952. 10.1071/FP05047 32689190

[kiaf521-B137] Sinclair TR, Hammond LC, Harrison J. Extractable soil water and transpiration rate of soybean on sandy soils. Agron J. 1998:90(3):363–368. 10.2134/agronj1998.00021962009000030008x

[kiaf521-B138] Sinclair TR, Jafarikouhini N, Pradhan D. Unexpectedly, triple super phosphate fertilizer induces maize drought resilience. J Plant Nutr. 2024:47(12):1906–1915. 10.1080/01904167.2024.2325948

[kiaf521-B139] Sinclair TR, Ludlow MM. Influence of soil water supply on the plant water balance of four tropical grain legumes. Aust J Plant Physiol. 1986:13:329–341. 10.1071/PP9860329

[kiaf521-B140] Sinclair TR, Messina CD, Beatty A, Samples M. Assessment across the United States of the benefits of altered soybean drought traits. Agron J. 2010:102(2):475–482. 10.2134/agronj2009.0195

[kiaf521-B141] Sinclair TR, Muchow RC. System analysis of plant traits to increase grain yield on limited water supplies. Agron J. 2001:93(2):263–270. 10.2134/agronj2001.932263x

[kiaf521-B142] Sinclair TR, Shekoofa A, Isleib TG, Balota M, Zhang H. Identification of Virginia-type peanut genotypes for water-deficit conditions based on early decrease in transpiration rate with soil drying. Crop Sci. 2018:58(6):2607–2612. 10.2135/cropsci2018.05.0293

[kiaf521-B143] Sinclair TR, Zwieniecki MA, Holbrook NM. Low leaf hydraulic conductance associated with drought tolerance in soybean. Physiol Plant. 2008:132(4):446–451. 10.1111/j.1399-3054.2007.01028.x 18333998

[kiaf521-B144] Skelton RP, Brodribb TJ, Choat B. Casting light on xylem vulnerability in an herbaceous species reveals a lack of segmentation. New Phytol. 2017:214(2):561–569. 10.1111/nph.14450 28124474

[kiaf521-B145] Subramanian KS, Charest C, Dwyer LM, Hamilton RI. Effects of arbuscular mycorrhizae on leaf water potential, sugar content, and P content during drought and recovery of maize. Can J Bot. 1997:75(9):1582–1591. 10.1139/b97-870

[kiaf521-B146] Tardieu F, Simonneau T, Muller B. The physiological basis of drought tolerance in crop plants: a scenario-dependent probabilistic approach. Annu Rev Plant Biol. 2018:69(1):733–759. 10.1146/annurev-arplant-042817-040218 29553801

[kiaf521-B147] Théroux-Rancourt G, Éthier G, Pepin S. Threshold response of mesophyll CO_2_ conductance to leaf hydraulics in highly transpiring hybrid poplar clones exposed to soil drying. J Exp Bot. 2014:65(2):741–753. 10.1093/jxb/ert436 24368507 PMC3904724

[kiaf521-B148] Thompson AJ, Andrews J, Mulholland BJ, McKee JMT, Hilton HW, Horridge JS, Farquhar GD, Smeeton RC, Smillie IRA, Black CR, et al Overproduction of abscisic acid in tomato increases transpiration efficiency and root hydraulic conductivity and influences leaf expansion. Plant Physiol. 2007:143(4):1905–1917. 10.1104/pp.106.093559 17277097 PMC1851808

[kiaf521-B149] Tolk JA, Howell TA, Evett SR. Nighttime evapotranspiration from alfalfa and cotton in a semiarid climate. Agron J. 2006:98(3):730–736. 10.2134/agronj2005.0276

[kiaf521-B150] Torres-Ruiz JM, Cochard H, Delzon S, Boivin T, Burlett R, Cailleret M, Corso D, Delmas CEL, De Caceres M, Diaz-Espejo A, et al Plant hydraulics at the heart of plant, crops and ecosystem functions in the face of climate change. New Phytol. 2024:241(3):984–999. 10.1111/nph.19463 38098153

[kiaf521-B151] Turner NC . Turgor maintenance by osmotic adjustment: 40 years of progress. J Exp Bot. 2018:69(13):3223–3233. 10.1093/jxb/ery181 29767758

[kiaf521-B152] Turner NC, Abbo S, Berger JD, Chaturvedi S, French RJ, Ludwig C, Mannur D, Singh S, Yadava H. Osmotic adjustment in chickpea (Cicer arietinum L.) results in no yield benefit under terminal drought. J Exp Bot. 2006:58(2):187–194. 10.1093/jxb/erl192 17088363

[kiaf521-B153] Tyree MT, Sperry JS. Vulnerability of xylem to cavitation and embolism. Annu Rev Plant Biol. 1989:40(1):19–36. 10.1146/annurev.pp.40.060189.000315

[kiaf521-B154] Vadez V . Root hydraulics: the forgotten side of roots in drought adaptation. Field Crops Res. 2014:165:15–24. 10.1016/j.fcr.2014.03.017

[kiaf521-B155] Vadez V, Grondin A, Chenu K, Henry A, Laplaze L, Millet EJ, Carminati A. Crop traits and production under drought. Nat Rev Earth Environ. 2024:5(3):211–225. 10.1038/s43017-023-00514-w

[kiaf521-B156] Vadez V, Kholova J, Medina S, Kakkera A, Anderberg H. Transpiration efficiency: new insights into an old story. J Exp Bot. 2014:65(21):6141–6153. 10.1093/jxb/eru040 24600020

[kiaf521-B157] Vadez V, Kholová J, Yadav RS, Hash CT. Small temporal differences in water uptake among varieties of pearl millet (Pennisetum glaucum (L.) R. Br.) are critical for grain yield under terminal drought. Plant Soil. 2013:371(1–2):447–462. 10.1007/s11104-013-1706-0

[kiaf521-B158] Velázquez-Márquez S, Conde-Martínez V, Trejo C, Delgado-Alvarado A, Carballo A, Suárez R, Mascorro JO, Trujillo AR. Effects of water deficit on radicle apex elongation and solute accumulation in Zea mays L. Plant Physiol Biochem. 2015:96:29–37. 10.1016/j.plaphy.2015.07.006 26218550

[kiaf521-B159] Vico G, Brunsell NA. Tradeoffs between water requirements and yield stability in annual vs. Perennial crops. Adv Water Resour. 2018:112:189–202. 10.1016/j.advwatres.2017.12.014

[kiaf521-B160] Volaire F . A unified framework of plant adaptive strategies to drought: crossing scales and disciplines. Glob Chang Biol. 2018:24(7):2929–2938. 10.1111/gcb.14062 29350812

[kiaf521-B161] Wang X, Huang J, Peng S, Xiong D. Leaf rolling precedes stomatal closure in rice (*Oryza sativa*) under drought conditions. J Exp Bot. 2023:74(21):6650–6661. 10.1093/jxb/erad316 37551729

[kiaf521-B162] Wang Y, Anderegg WR, Venturas MD, Trugman AT, Yu K, Frankenberg C. Optimization theory explains nighttime stomatal responses. New Phytol. 2021:230(4):1550–1561. 33576001 10.1111/nph.17267

[kiaf521-B163] Wankmüller FJP, Carminati A. Stomatal regulation prevents plants from critical water potentials during drought: result of a model linking soil–plant hydraulics to abscisic acid dynamics. Ecohydrology. 2022:15(5):e2386. 10.1002/eco.2386

[kiaf521-B164] Wong SC, Cowan IR, Farquhar GD. Stomatal conductance correlates with photosynthetic capacity. Nature. 1979:282(5737):424–426. 10.1038/282424a0

[kiaf521-B165] Zaman-Allah M, Jenkinson DM, Vadez V. A conservative pattern of water use, rather than deep or profuse rooting, is critical for the terminal drought tolerance of chickpea. J Exp Bot. 2011:62(12):4239–4252. 10.1093/jxb/err139 21610017 PMC3153682

