# Supplementary material for: Plant hydraulic traits influencing crop production in water-limited environments
Source: Plant Physiol. 2025 Oct 14;199(3):kiaf521. doi: 10.1093/plphys/kiaf521 (PMC12596301; doi:10.1093/plphys/kiaf521)
Supplement: kiaf521_Supplementary_Data [file kiaf521_supplementary_data.zip › Supplementary Figure 1.docx]

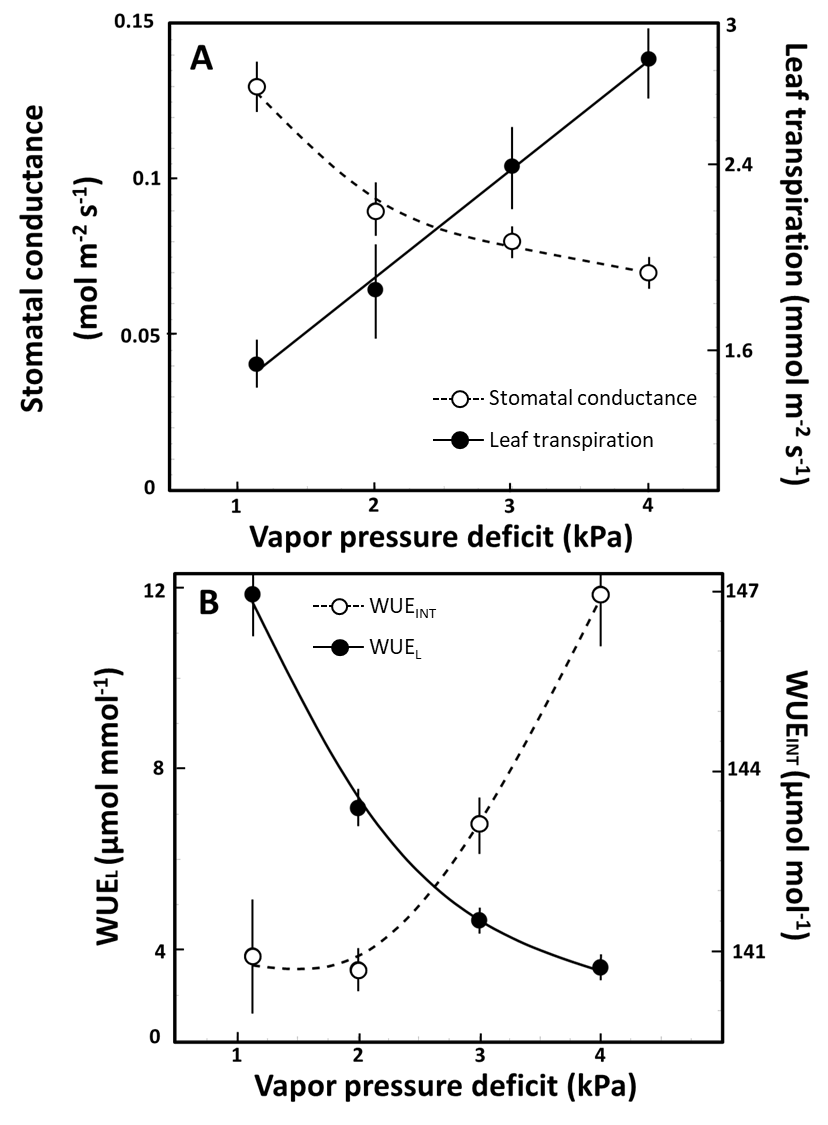


**Supplementary Figure 1.** Gas exchange and water use efficiency (WUE) of cotton leaves with increases in vapor pressure deficit (VPD). (A) Concomitant increases in transpiration (closed circles) and decreases in stomatal conductance (open circles) with increases in VPD. (B) Contrasting responses of leaf WUE (WUE_L_ = photosynthesis/transpiration) (closed circles) and intrinsic WUE (WUE_INT_ = photosynthesis/stomatal conductance) (open circles) with increases in VPD. Because of the opposite behavior of stomatal conductance and transpiration with increases in VPD (A), concomitant increases in WUE_INT_ and decreases in WUE_L_ are typically observed (B). Therefore, many authors normalize WUE_L_ to a common VPD or simply use WUE_INT_ when comparing the WUE among genotypes (Leakey et al., 2019). Data are means ± standard error (n = 5). Original data can be found in the Supplementary Dataset. Data were obtained by Andrade and Cardoso (unpublished results).
